# Functional and Disability Outcomes in NSCLC Patients Post-Lobectomy Undergoing Pulmonary Rehabilitation: A Biopsychosocial Approach

**DOI:** 10.3390/cancers16122281

**Published:** 2024-06-20

**Authors:** Agnieszka Zawadzka-Fabijan, Artur Fabijan, Mariusz Łochowski, Łukasz Pryt, Bartosz Polis, Krzysztof Zakrzewski, Jolanta Ewa Kujawa, Józef Kozak

**Affiliations:** 1 Department of Medical Rehabilitation, Faculty of Health Sciences, Medical University of Lodz, 90-419 Lodz, Poland; jolanta.kujawa@umed.lodz.pl; 2 Department of Neurosurgery, Polish-Mother’s Memorial Hospital Research Institute, 93-338 Lodz, Poland; artur8944@wp.pl (A.F.); jezza@post.pl (B.P.); krzysztof.zakrzewski@iczmp.edu.pl (K.Z.); 3 Clinic of Thoracic Surgery and Respiratory Rehabilitation, Regional Multi-Specialist Center for Oncology and Traumatology of the Nicolaus Copernicus Memorial Hospital in Lodz, 93-513 Lodz, Poland; marilo@op.pl (M.Ł.); lpryt@mp.pl (Ł.P.); sekretariat.torakochirurgii@kopernik.lodz.pl (J.K.)

**Keywords:** ICF, lung cancer, NSCLC, lung lobectomy, WHODAS 2.0

## Abstract

**Simple Summary:**

Lung cancer is the foremost cause of cancer incidence and mortality on a global scale, and effective treatment often involves surgical removal of lung tissue. This study aimed to create a comprehensive version of the International Classification of Functioning, Disability and Health (ICF) tailored for patients with lung cancer after thoracic surgery undergoing pulmonary rehabilitation. By assessing patients’ functional profiles and levels of disability using this comprehensive ICF Core Set and the WHO Disability Assessment Schedule (WHODAS 2.0), the research sought to better understand the impact of lung cancer and its treatment on daily life. The findings suggest that the tailored ICF tool effectively captures the functional status of these patients, highlighting its potential utility in improving clinical practice and patient rehabilitation. This research may significantly impact the medical community by providing a comprehensive framework for evaluating and managing the functional health of lung cancer patients post-surgery.

**Abstract:**

Worldwide, lung cancer remains the predominant cause of cancer cases and deaths and poses significant health challenges, with surgical resection being a key treatment. Post-surgery, patients often experience functional impairments. This study aimed to develop a comprehensive ICF version for assessing the functional profile and disability in lung cancer patients post-thoracic surgery undergoing pulmonary rehabilitation using the ICF and WHODAS 2.0 tool. We analyzed the correlation between the ICF Core Set and WHODAS 2.0 data to understand the impact on daily functioning. This study included 50 patients (23 F, 27 M) from the Clinic of Thoracic Surgery and Respiratory Rehabilitation in Lodz. Essential ICF codes were determined using the Delphi method, and assessments were conducted on the third day post-operation. Statistical analyses included various tests with α = 0.05. The results showed no impairments in voice functions (b310), respiration rates (b4400), and diaphragm functions (b4451), but there were significant issues with chest pain (b28011), respiratory muscle functions (b445), exercise tolerance (b455), and muscle endurance (b740). In Activities and Participation and Environmental Factors, most codes were not problematic, except for employment (d845, d850) and atmospheric pressure (e2252). Significant correlations were found between mobility limitations (d410, d460) and self-care (d510, d540) with the WHODAS 2.0 results. The comprehensive ICF Core Set effectively described the functional profile of post-surgery patients, confirming its utility and highlighting the impact of disability on daily functioning.

## 1. Introduction

Lung cancer constitutes the primary cause of cancer incidence and death worldwide, accounting for 1.8 million deaths annually and 2.2 million new cases reported in 2020. It is the leading cause of cancer incidence and mortality among men and ranks third in incidence and second in mortality among women [1]. Clinically, lung cancer is classified into two primary types: non-small cell lung cancer (NSCLC) and small cell lung cancer (SCLC), accounting for approximately 85–90% and 10–15% of all lung cancer cases, respectively [2,3].

The development of lung cancer is strongly associated with environmental factors and lifestyle choices, with tobacco smoking recognized as the primary causative factor [4]. Other significant risk factors include exposure to asbestos, chromium, arsenic, radon, and polycyclic aromatic hydrocarbons [5,6]. Although smoking accounts for over 80% of lung cancer cases, approximately 10–15% of diagnoses occur in individuals who have never smoked [7]. The long-term prognosis of patients with early-stage NSCLC is reportedly satisfactory, with 5-year survival rates after resection of over 70% [8,9].

Surgical treatment is the main therapeutic strategy for lung cancer patients and is typically considered the first-line treatment for patients with early-stage NSCLC who are healthy enough to undergo surgery [10]. While a lobectomy is the most commonly performed surgical procedure for early-stage lung cancer, it is not always necessary. Many patients today are treated with segmental resection and, in some specific cases, after induction with immunotherapy [11,12].

The International Classification of Functioning, Disability and Health (ICF), developed by the World Health Organization (WHO), is a universal language for describing and measuring health and health-related states. It allows for a comprehensive description of a patient’s situation, considering multiple factors affecting their health. It also includes environmental and personal factors in the assessment, categorized as contextual factors. This enables a more holistic evaluation of the impact of health on daily functioning, taking into account both biological and psychosocial factors. Over the years, numerous brief and comprehensive ICF Core Sets have been developed for various diseases; however, a dedicated set for lung cancer patients is still lacking [13]. 

As part of the ongoing development of the biopsychosocial model of disability, the WHO created another tool, WHODAS 2.0, which measures health and disability in adults at the population level or in clinical practice. WHODAS 2.0 assesses average functioning in daily situations over the past 30 days and examines six domains of functioning: (1) cognition, (2) mobility, (3) self-care, (4) getting along with others, (5) life activities, and (6) participation in society. The most widespread and validated form of WHODAS 2.0 is the 36-item interview version, which takes about 20 min to complete and has excellent psychometric properties [14,15]. The ICF and WHODAS 2.0 serve as global standards for assessing health and disability, which is crucial in evaluating the impact of lung cancer on patients’ daily functioning [13,15].

Surgery for lung cancer patients significantly impacts their quality of life, disability level, and functional profile [16,17]. Assessing these parameters can aid in better understanding the full impact of surgical intervention, enhancing patient–surgeon communication, improving patient counseling and expectation setting, and monitoring treatment and therapeutic progress.

The aim of this study was to create a comprehensive ICF Core Set dedicated to patients with lung cancer after thoracic surgery undergoing pulmonary rehabilitation. This study focused on assessing the functional profile and disability level of this patient group using the ICF and the WHODAS 2.0 questionnaire. Additionally, this study analyzed the correlation between the results obtained from the comprehensive ICF Core Set (*Activity and Participation* domain), and the data from WHODAS 2.0, hypothesizing that it would provide a deeper understanding of the impact of lung cancer on patients’ daily functioning post-surgery. The hypotheses presented in this study are as follows:

**Hypothesis** **1** **(H1):**  *The comprehensive ICF Core Set for patients with lung cancer after thoracic surgery undergoing pulmonary rehabilitation enables a comprehensive description of the patient* *’s functional profile.*

**Hypothesis** **2** **(H2):**  *The disability level assessed using the WHODAS 2.0 questionnaire correlates with the Activity and Participation domain of the comprehensive ICF Core Set for patients with lung cancer after thoracic surgery undergoing pulmonary rehabilitation.*

## 2. Materials and Methods

### 2.1. Study Design

This study was conducted at the Clinic of Thoracic Surgery and Respiratory Rehabilitation at the Regional Multi-Specialist Center for Oncology and Traumatology of the Nicolaus Copernicus Memorial Hospital in Lodz. The project received approval from the Bioethics Committee of the Medical University of Lodz (RNN/183/15/KB). The scientific study did not interfere with the standard therapeutic process of the patients. 

### 2.2. Participants

Patients were enrolled in this study at the Clinic of Thoracic Surgery and Respiratory Rehabilitation at the Regional Multi-Specialist Center for Oncology and Traumatology of the Nicolaus Copernicus Memorial Hospital in Lodz between 2016 and 2020 (*n* = 50; 23 F, 27 M). This study included individuals over 18 years old who were eligible for a surgical lobectomy due to early-stage non-small cell lung cancer (NSCLC), which was performed using standard posterolateral thoracotomy under general anesthesia with a double-lumen tube, and had no contraindications for postoperative rehabilitation and no postoperative complications. All patients provided informed consent to participate in this study. Inclusion criteria encompassed patients over 18 years old who provided informed consent and were qualified for a posterior thoracotomy lobectomy due to stage I or II NSCLC. Exclusion criteria included individuals under 18 years old, those who did not provide informed consent, patients not qualified for a lobectomy or qualified for reasons other than NSCLC, surgeries performed using techniques other than posterolateral or video-assisted thoracic surgery (VATS), patients operated on in stages other than I or II, individuals undergoing reoperation, patients with severe medical issues preventing postoperative rehabilitation, and individuals with intellectual disabilities preventing consent or cooperation with the therapist post-surgery.

### 2.3. Instruments and Measures

#### 2.3.1. Development of a Literature Review and Selection of ICF Categories

The consensus Delphi method was used to determine the essential ICF codes for creating a comprehensive ICF Core Set for patients with lung cancer after thoracic surgery undergoing pulmonary rehabilitation. A literature review was conducted to list all potential factors associated with lung cancer and common patient problems. These were then linked to relevant ICF codes, forming a proposed set of codes. Three rounds of surveys were conducted among twenty-one specialists in the field (thoracic surgeons, rehabilitation specialists, nurses, and physiotherapists working with these patients) to confirm the most relevant codes. Articles for the review were searched using keywords such as ‘lung cancer’, ‘NSCLC’, ‘quality of life’, ‘lung lobectomy’, and ‘pulmonary rehabilitation’. All of the relevant literature was included in a further assessment, during which the methodological quality was analyzed. Selected articles were independently reviewed by two reviewers to identify factors related to the quality of life in lung cancer. In case of discrepancies between reviewers, a third reviewer decided whether a factor should be included. All significant factors were then defined and linked to ICF codes, with 101 codes identified for further evaluation. 

#### 2.3.2. Consensus Procedure

**Figure 1:**
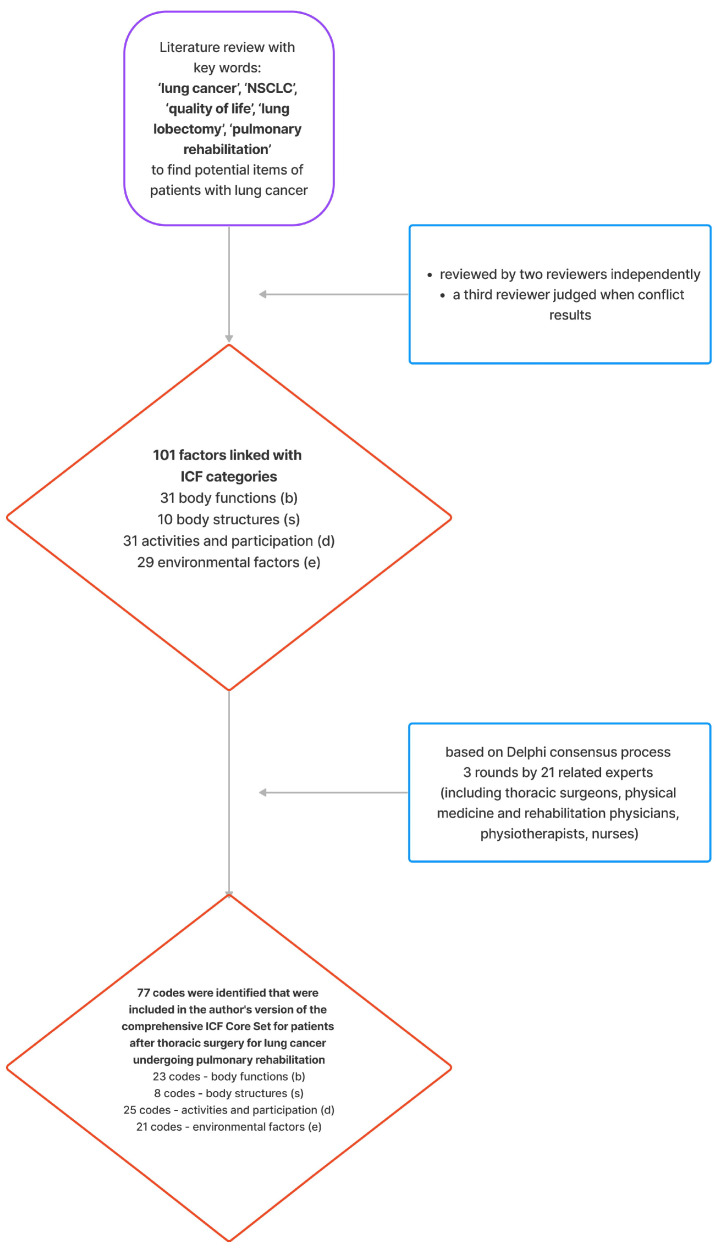
Study flowchart.

The selected ICF codes were evaluated in three formal Delphi consensus rounds [18]. In each round, participants were asked to rate the importance of the selected codes, indicating whether they wanted the code included in the final set (YES or NO). Results for each code in the first round served as reference data for the second round. Based on this information, all participants were asked to reassess their previous answers, average scores of all participants, and standard deviations. Each item was reassessed in the second round. Similarly, results for each item in the second round served as reference data for the third round. The consensus-building process concluded after 3 rounds of surveys, ultimately identifying 77 codes to be included in the comprehensive ICF Core Set (Figure 1).

#### 2.3.3. A Comprehensive ICF Core Set Specifically Designed to Capture the Functional Impairments and Limitations of Lung Cancer Patients

**Figure 2:**
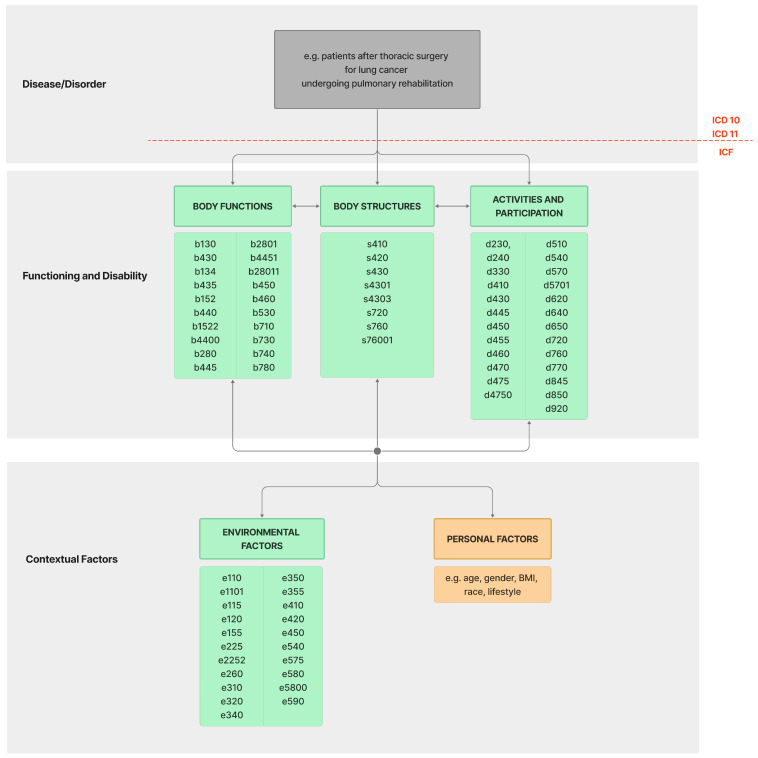
A Comprehensive ICF Core Set specifically designed to capture the functional impairments and limitations of lung cancer patients—77 codes.

On the third day after surgery, the functional profile of patients was assessed using a comprehensive version of the ICF Core Set for lung cancer (77 codes at the second, third, and fourth levels) consisting of the *Body Functions* domain—23 codes: b130, b134, b152, b1522, b280, b2801, b28011, b310, b410, b430, b435, b440, b4400, b445, b4451, b450, b455, b460, b530, b710, b730 b740, and b780; the *Body Structures* domain—8 codes: s410, s420, s430, s4301, s4303, s720, s760, and s76001; the *Activities and Participation* domain—25 codes: d230, d240, d330, d410, d430, d445, d450, d455, d460, d470, d475, d4750, d510, d540, d570, d5701, d620, d640, d650, d720, d760, d770, d845, d850, and d920; and the *Environmental Factors* domain—21 codes: e110, e1101, e115, e120, e155, e225, e2252, e260, e310, e320, e340, e350, e355, e410, e420, e450, e540, e575, e580, e5800, and e590 (Figure 2). 

The ICF domains require the use of one or more qualifiers to assess specific codes. 

The analysis of the *Body Function* domain codes involved the quantitative assessment of a single qualifier indicating the extent or magnitude of impairment according to the ICF methodology described in Table 1. 

The analysis of the *Body Structures* domain codes involved assessing three qualifiers, the *Extent*, *Nature*, and *Localization* of an impairment. The first qualifier, *Extent*, is used to determine the degree of impairment (Table 2).

The second qualifier, *Nature*, is used to determine the nature of the change in the relevant body structure. The third qualifier, *Localization*, is used to specify the precise location of the given body structure (Table 3).

For statistical analysis and the creation of a functional profile for the *Body Structures* domain, data from the first qualifier were used. 

The analysis of codes in the *Activities and Participation* domain involved assessing two qualifiers: *Performance* and *Capacity* (Table 4). The *Performance* qualifier describes what the individual has performed in their current environment, while the *Capacity* qualifier describes the individual’s ability to perform a given task or activity.

The analysis of codes in the *Environmental Factors* domain involved the quantitative assessment of a single qualifier (Table 5). This qualifier indicates to what extent an environmental factor serves as a *Facilitator* or *Barrier* [13].

#### 2.3.4. The World Health Organization Disability Assessment Schedule (WHODAS 2.0)

Also, on the third postoperative day, the 36-item version of the WHODAS 2.0 questionnaire was used to assess the degree of disability. The 36-item version is the most detailed. This tool provides information on the level of functioning and degree of disability in six domains of life as follows:

- Domain 1: Cognition (D1)—understanding and communicating;
- Domain 2: Mobility (D2)—moving and getting around;
- Domain 3: Self-care (D3)—e.g., hygiene, dressing, and eating;
- Domain 4: Getting along (D4)—assesses interactions with other people and difficulties that may arise in this area of life due to health status;
- Domain 5: Life activities (D5)—household and work/school activities;
- Domain 6: Participation (D6)—assesses social dimensions. The questions do not necessarily refer exclusively to the ICF component of participation but also consider various contextual factors (personal and environmental) affecting the respondent’s health status [14,15].

The analysis of responses from individual questions in the domains (D1, D2, D3, D4, D5, and D6) involved a quantitative assessment according to the methodology described in Table 6.

Additionally, for each item with a positive response, respondents were asked about the number of days (within the last 30 days) they experienced the specified difficulty. The 36-item version is available in 3 different formats: interviewer-administered, self-administered, or administered by a proxy (e.g., family member, caregiver). In this study, the interviewer-administered 36-item version was used. The average interview duration was 20 min.

#### 2.3.5. Rehabilitation Procedure Scheme

Preoperative rehabilitation of patients began immediately upon hospital admission, focusing on psychological and physical preparation for the intensive stress of surgery. Postoperative rehabilitation continued from the first day after surgery until the day of discharge, with a rehabilitation program implemented twice daily, and each physiotherapy session lasted approximately 30 min. Before and after the exercises, the physiotherapist checked the proper connection of the drains and the patient’s overall condition. The program included diaphragmatic and resistance breathing exercises using trainers, stabilization exercises around the postoperative wound, effective coughing exercises with percussion techniques, postural drainage, thromboprophylaxis exercises, and general mobility exercises to improve shoulder girdle and chest mobility, as well as gradual mobilization. Due to the nature of this study, no control group was involved.

#### 2.3.6. Data Analysis

The significance level of the statistical tests in this analysis was set at α = 0.05. Distributions for categorical variables were described by calculating the frequencies (*n*) and the corresponding percentages (%) for each category. To determine the statistical significance of the differences between two independent groups for categorical variables, Pearson’s chi-squared test, Fisher’s exact test, and a proportion test were conducted.

A comparison of the medians between two independent groups with the non-parametric distribution of variables was performed using the Wilcoxon rank-sum test. To analyze the relationships between various dimensions of the questionnaires, Spearman’s rank correlation coefficient (*rho*) was used. Associated *p*-values were calculated using the asymptotic approximation to the t-distribution.

#### 2.3.7. Statistical Environment

Analyses were conducted using the R statistical language (version 4.3.1 [19]) on a Windows 10 Pro 64-bit system (build 19045), using the *report* (version 0.5.7 [20]), *gtsummary* (version 1.7.2 [21]), *readxl* (version 1.4.3 [22]), *dplyr* (version 1.1.3 [23]), and *psych* (version 2.3.9 [24]) packages.

## 3. Results

### 3.1. Characteristics of the Sample

This study, conducted to analyze the functional profile of patients on the third day post-lobectomy for lung cancer, included a sample of 50 individuals. The demographic composition of the sample was nearly equally distributed by gender: 46% of the participants were female (*n* = 23), while males constituted 54% of the sample (*n* = 27). The mean age of the patients at the time of this study was 62.0 years, with a standard deviation (SD) of 11.4 years. The age range of this study population was broad, spanning from 27 to 78 years, indicating significant heterogeneity in the age of the participants.

### 3.2. Characteristics of Sociodemographic Parameters, Clinical Data, and Major Risk Factors of the Studied Sample

To understand the patient profile, identify potential prognostic correlations, and tailor individualized treatment and care strategies, a review and analysis of sociodemographic parameters, clinical data, and major risk factors were performed. The dataset includes demographic variables, such as age and gender, as well as detailed clinical data and selected risk factors deemed relevant in the context of describing patients with lung cancer. Analyzing these data allows for the identification of potential differences in disease progression and treatment response, which is crucial for optimizing multifaceted therapeutic and treatment strategies. Table 7 presents the detailed characteristics of the study sample, including the gender distribution of the subjects.

The age of the patients in the entire sample was varied, with a median, *Mdn* (Q1, Q3), of 60 years (56.50, 65.00) for women and 65 years (61.00, 70.00) for men. The age difference between genders was only significant at the trend level (*p* = 0.070).

Analysis of smoking history data revealed that 34 patients (68%) in the entire sample were smokers. Among women, this percentage was 56.52% (13 individuals), whereas, among men, it was 77.78% (21 individuals). Differences in smoking prevalence between genders were not statistically significant (*p* = 0.108).

The body mass index (BMI) of patients, averaging 24.60 kg/m^2^, was within the normal range, with slight gender differences (24.80 kg/m^2^ for women and 24.28 kg/m^2^ for men), which were not statistically significant (*p* = 1.00).

The location of cancerous lesions in the lungs was evenly distributed between the left and right lung (50% of cases each). However, there was a trend towards more frequent occurrence of lesions in the left lung among men (62.96%) and in the right lung among women (65.22%), which was statistically significant (*p* = 0.047).

The lung re-expansion time, an important indicator in the recovery process post-lobectomy, averaged 5 days (Q1, Q3: 4.00, 5.00 days) across the sample, with minor gender differences that were not statistically significant (*p* = 0.265).

A history of previous cancer episodes was present in 17 patients (34%), with a higher frequency among women (43.48%) than men (25.93%), which was also not statistically significant (*p* = 0.192).

In summary, there was a diversity of risk factors and clinical characteristics among patients undergoing a lung lobectomy by gender. Notably, the statistically significant difference in the location of cancerous lesions relative to gender could influence diagnostic and therapeutic approaches. Differences in smoking prevalence and previous cancer episodes between genders, despite the lack of statistical significance, may have clinical importance and require further analysis in the context of risk factors and prognosis.

### 3.3. Assessment of Inter-Rater Agreement among Experts on Individual ICF Codes Using the Delphi Consensus Method

Within the Delphi process, a multi-step method aimed at achieving consensus among experts, and the proprietary set of the ICF codes was evaluated. Assessing the level of agreement among raters is a crucial element that allowed us to determine whether and to what extent experts agree to include specific code proposals in the final version of the comprehensive ICF Core Set for lung cancer patients. Table 8, Table 9, Table 10 and Table 11 present the consensus ratings from the Delphi process for the selected components of the ICF Core Set: *Body Functions*, *Body Structures*, *Activities and Participation,* and *Environmental Factors.* For each code, the definition and the degree of agreement among raters, expressed as the mean (M) and standard deviation (SD), are shown across the three rounds of this study. Agreement is presented as a numerical value, where 1.00 ± 0.00 indicates full agreement, and values approaching 0 indicate a lack of agreement among experts.

The tables include codes for the following classification components: *Body Functions* (Table 8), *Body Structures* (Table 9), *Activities and Participation* (Table 10), and *Environmental Factors* (Table 11). The presented data illustrate the evolution of consensus over successive rounds of the Delphi method, allowing for the observation of the dynamics of changes in agreement among raters.

### 3.4. Characteristics of the Results for Individual Codes of the Comprehensive ICF Core Set for Lung Cancer

#### 3.4.1. Domain—Body Functions

The comparative analysis of the *Body Function* domain between women and men in the studied sample revealed several significant differences, as well as many similarities. 

One of the most notable differences was observed in the *Energy and drive functions (b130).* A significantly higher percentage of men (33.33%) compared to women (4.35%) showed no impairment in this category (*p* = 0.011). Simultaneously, a higher percentage of women (21.74%) compared to men (7.41%) experienced moderate impairment, although this difference did not reach statistical significance (*p* = 0.146).

In other codes, no statistically significant differences between genders were found. However, certain trends were observed. In the code *Heart functions (b410)*, a higher percentage of men (55.56%) compared to women (26.09%) showed no impairment, while more women (43.48%) than men (18.52%) experienced mild impairment. These differences, however, were not statistically significant (*p* = 0.115).

Similarly, in the code *Additional respiratory functions (b450),* a trend was observed where a higher percentage of men (11.11%) compared to women (0.00%) showed no impairment, while a higher percentage of women (56.52%) compared to men (37.04%) experienced mild impairment. These differences also did not reach statistical significance (*p* = 0.189).

For other codes, such as *Sleep functions (b134)*, *Emotional functions (b152)*, *Sensation of pain (b280)*, *Voice functions (b310)*, *Hematological system functions (b430)*, *Immunological system functions (b435)*, *Respiration functions (b440)*, *Exercise tolerance functions (b455)*, *Sensations associated with cardiovascular and respiratory functions (b460)*, *Weight maintenance functions (b530)*, *Mobility of joint functions (b710)*, *Muscle power functions (b730)*, *Muscle endurance functions (b740),* and *Sensations related to muscles and movement functions (b780)*, the distribution of impairment levels was similar between both genders, and the differences did not reach statistical significance (Supplementary Material Table S1).

#### 3.4.2. Domain—Body Structures

To assess the impact of gender on individual codes in the *Body Structures* domain, a comparative analysis between women and men in the studied sample was conducted (Supplementary Material Table S2). The following table presents the results of the evaluation by patients of the first qualifier describing the level of impairment for individual domain codes. 

A significant difference was observed in the code *Structure of the cardiovascular system (s410)*. A significantly higher percentage of men (55.56%) compared to women (26.09%) showed no impairment in this category (*p* = 0.035). Simultaneously, a higher percentage of women (43.48%) compared to men (18.52%) presented mild impairment, although this difference did not reach statistical significance (*p* = 0.055). Additionally, a higher percentage of women (26.09%) compared to men (7.41%) experienced moderate impairment, but this difference was also not statistically significant (*p* = 0.072).

Another code within the *Body Structures* domain, showing significant differences, was the *Thoracic vertebral column (s76001).* The vast majority of men (96.30%) showed no impairment in this code, while this percentage among women was 82.61%. However, this difference did not reach statistical significance (*p* = 0.351). Simultaneously, a higher percentage of women (17.39%) compared to men (0.00%) presented mild impairment, although this difference was also not statistically significant (*p* = 0.239).

For other codes, such as the *Structure of the immune system (s420)*, the *Structure of the respiratory system (s430)*, *Lungs (s4301)*, *Muscles of respiration (s4303)*, the *Structure of the shoulder region (s720),* and the *Structure of the trunk (s760)*, the distribution of impairment levels was similar between both genders, and the differences did not reach statistical significance.

Noteworthy is the trend observed in the code *Lungs (s4301)*, where a higher percentage of men (51.85%) compared to women (30.43%) showed severe impairment, while a higher percentage of women (65.22%) compared to men (48.15%) presented moderate impairment. These differences, however, did not reach statistical significance (*p* = 0.194).

#### 3.4.3. Domain—Activities and Participation Qualifiers, Performance, and Capacity

Assessing the impact of gender on patient functioning, the evaluation results of the codes within the *Activities and Participation* domain qualifiers, *Participation*, and *Capacity*, were analyzed in the studied sample (Supplementary Material Tables S3 and S4). 

The analysis showed no statistically significant differences between women and men in most of the evaluated codes. Specifically, in the code *Carrying out daily routine (d230),* a higher percentage of men (29.63%) than women (8.70%) reported no difficulty, but this difference was not statistically significant. Similarly, in the code *Handling stress and other psychological demands (d240),* both women and men most often reported mild difficulty, with no significant differences between genders.

In the code *Speaking (d330)*, the majority of participants, both women (82.61%) and men (77.78%), had no difficulty speaking. However, some men reported moderate and complete difficulty. In the code *Changing basic body position (d410)*, the vast majority of both women (86.96%) and men (81.48%) reported no difficulty, with no statistically significant differences.

The code *Lifting and carrying objects (d430)* also showed no significant gender differences, with most participants reporting mild difficulty. Similarly, in the code *Hand and arm use (d445)*, both women (65.22%) and men (51.85%) most frequently reported no difficulty, with no significant differences.

In the code *Walking (d450)*, all female participants and most male participants (92.59%) reported no difficulty. Additionally, in the code *Moving around (d455)*, all respondents considered this activity as not applicable, indicating its lack of relevance in daily functioning.

The code *Moving around in different locations (d460)* showed some differences, with women more often reporting no difficulty (82.61%) compared to men (62.96%), but this difference was not statistically significant. In the code *Using transportation (d470),* a higher percentage of men (29.63%) compared to women (21.74%) reported no difficulty, but these differences were not statistically significant.

In the codes *Driving (d475)* and *Driving human-powered transportation (d4750),* gender differences were minimal and not statistically significant. In the code *Washing oneself (d510)*, men slightly more frequently reported no difficulty, but these differences were also not significant.

In the code *Dressing* (d540), both women (78.26%) and men (81.48%) most frequently reported mild difficulty, with negligible differences. Similarly, in the codes *Looking after one’s health (d570)* and *Managing diet and fitness (d5701)*, no significant gender differences were observed, although women slightly more often reported moderate difficulty in managing diet and physical condition.

The code *Acquisition of goods and services (d620)* indicated that most participants, regardless of gender, had no difficulty acquiring goods and services. In the code *Doing housework (d640)*, women more often reported mild difficulty, but the differences were not statistically significant. Similarly, in the code *Caring for household objects (d650)*, the ratings were similar for both genders.

In codes related to interpersonal relationships, such as *Complex interpersonal interactions (d720)* and *Family relationships (d760)*, all participants reported no difficulty. In the code *Intimate relationships (d770)*, women more often reported no difficulty (91.30%) compared to men (74.07%), but this difference was not statistically significant.

In the codes *Acquiring, keeping, and terminating a job (d845)* and *Remunerative employment (d850),* no significant gender differences were observed, although a higher percentage of women reported no difficulty. In the code *Recreation and leisure (d920)*, women more often reported mild difficulty (52.17%), while men more frequently reported moderate or severe difficulty, but these differences were also not statistically significant.

In summary, the analysis of the ICF questionnaire results showed no statistically significant differences between women and men in most codes in the *Activities and Participation* domain. The results suggest that the level of difficulty in daily functioning is similar for both genders, emphasizing the importance of an individualized approach to the assessment and rehabilitation of patients undergoing thoracic surgery for lung cancer.

#### 3.4.4. Domain—Environmental Factors

Supplementary Material Table S5 presents the characteristics of the *Environmental Factors* domain in the studied sample, divided by patient gender. The aim of the analysis was to compare the impact of various environmental factors on the functioning of women and men.

In the code *Products or substances for personal consumption (e110)*, a majority of the ratings indicated a complete or substantial facilitator for both women (34.78% and 43.48%) and men (44.44% and 22.22%), although these differences did not reach statistical significance (*p* = 0.375).

Similarly, in the code *Drugs (e1101)*, the ratings of the complete facilitator predominated (78.26% of women and 81.48% of men), with no significant differences between genders (*p* = 1.00).

The assessment of codes, such as *Products and technology for personal use in daily living (e115)* and *Products and technology for personal indoor and outdoor mobility and transportation (e120),* also showed no significant differences between women and men. In both cases, the ratings of the complete or substantial facilitator predominated, with a slight predominance of complete facilitator among men (44.44% vs. 34.78% of women for e115; 44.44% vs. 34.78% of women for e120).

In the codes *Immediate family (e310)*, *Friends (e320),* and *Health professionals (e355),* the ratings mainly indicated a complete or substantial facilitator, with no significant differences between genders.

Similarly, *Individual attitudes of immediate family members* (e410), *Individual attitudes of friends* (e420), and *Individual attitudes of health professionals (e450)* were mostly rated as substantial or moderate facilitators, regardless of gender.

The analysis of codes *Transportation services, systems, and policies (e540)*, *General social support services*, *systems, and policies (e575)*, *Health services, systems, and policies (e580),* and *Labor and employment services*, *systems, and policies (e590)* also revealed no significant differences between women and men. Ratings in these codes varied, including facilitators of varying degrees and occasionally barriers, but there was no clear gender-related trend.

In summary, the analysis of the *Environmental Factors* domain did not reveal significant differences between women and men in the studied sample. Ratings of individual codes were similar for both groups, mainly indicating facilitators of varying degrees, with rare cases of barriers. The results suggest that gender is not a significant differentiating factor in the perception of environmental factors in the studied population.

### 3.5. Average Results for Individual Codes of the Comprehensive ICF Core Set for Lung Cancer

Analyzing the average patient results, we can observe that the study group in the *Body Function* domain did not show impairments in codes *Voice functions (b310)*, *Respiration rate (b4400),* and *Functions of the diaphragm (b4451).* In other codes, mild or moderate impairment was observed. Severe impairment included *Sensation of pain (b280)*, particularly *Pain in the chest (b28011)*, and *Respiration functions (b440)*, specifically *Respiratory muscle functions (b445)*, *Exercise tolerance functions (b455*), and *Muscle endurance functions (b740).*

In the *Body Structures* domain, patients reported no impairment in the codes *Structure of the trunk (s760)* and *Thoracic vertebral column (s76001).* Other codes related to the cardiovascular, immune, and respiratory systems showed mild or moderate impairment. Severe impairment was observed in the code *Structure of respiratory system (s430)* and second-level codes *Lungs (s4301)* and *Muscles of respiration (s4303).*

In the *Activity and Participation* domain, codes that respondents indicated no difficulty in both the *Participation* and *Capacity* qualifiers included *Speaking (d330)*, *Changing basic body position (d410)*, *Hand and arm use (d445)*, *Walking (d450)*, *Moving around in different locations (d460)*, *Acquisition of goods and services (d620)*, *Complex interpersonal interactions (d720)*, *Family relationships (d760)*, *Intimate relationships (d770)*, and *Recreation and leisure (d920)*. The code *Moving around (d455)* was generally considered not applicable. Other codes were rated as mild or moderate difficulty. The worst ratings were for codes related to employment, such as *Acquiring*, *keeping, and terminating a job (d845)* and *Remunerative employment (d850),* with a predominance of difficulty in the *Performance* qualifier. 

**Figure 3:**
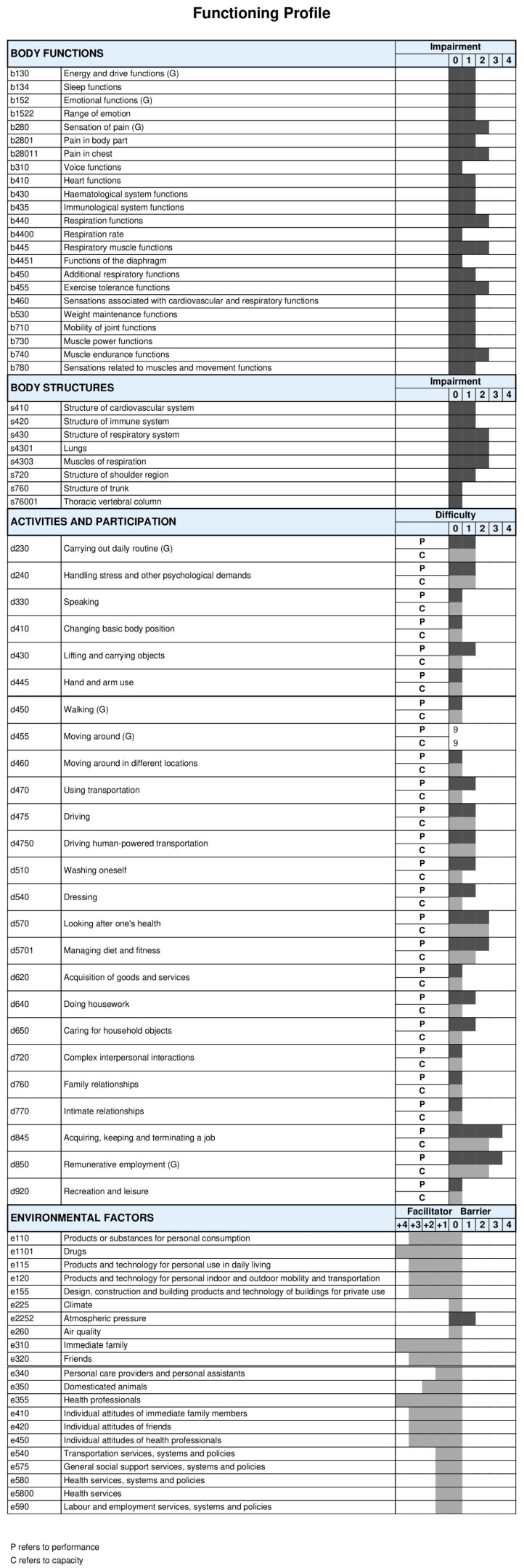
Functioning profile of the patients after thoracic surgery for lung cancer undergoing pulmonary rehabilitation. The ICF codes used to create the profile of functioning of this study sample represent the codes that have averaged within each ICF category. This profile of functioning was built while using the ICF-based documentation form on the following web page, https://www.icf-core-sets.org/en/page0.php accessed on 26 April 2024, courtesy of the ICF Research Branch.

In the *Environmental Factors* domain, only the code, *Atmospheric pressure (e2252),* was rated as a mild barrier. Other codes were considered facilitators by the respondents. Complete facilitators included *Drugs (e1101)*, *Immediate family (e310),* and *Health professionals (e355)* (Figure 3).

### 3.6. Characteristics of the Results of Individual Categories of the WHODAS 2.0 Questionnaire

The table below presents the results of the WHODAS 2.0 disability assessment questionnaire, divided by patient gender (Table 12). This questionnaire assesses the degree of disability across six domains: *Cognition (D1)*, *Mobility (D2)*, *Self-care (D3)*, *Getting along (D4)*, *Life activities (D5)*, and *Participation (D6)*.

For each question within the respective domains, the distribution of responses (1—none, 2—mild, 3—moderate, 4—severe, 5—extreme or cannot do) is presented separately for women and men. 

The analysis of data from the WHODAS 2.0 questionnaire indicates certain differences in the level of functioning between women and men in the studied patient group, although, in most domains, these differences did not reach statistical significance.

In the *Cognition (D1)* domain, no statistically significant differences between genders were found. Both women and men mostly reported no problems or only mild difficulties with concentration, memory, problem solving, learning, and understanding speech.

Similarly, in the *Mobility (D2)*, *Self-care (D3)*, and *Getting along (D4)* domains, the differences between women and men were small and not statistically significant. Patients of both genders most often rated their limitations in these domains as none or mild.

The only statistically significant difference (*p* = 0.031) was observed in the question *Making new friends? (D4.4)*, where a higher percentage of men (37%) than women (8.7%) reported no difficulty in this aspect.

In the *Life activities (D5)* domain, women more often reported no problems in performing household tasks and responsibilities, although the differences were not significant. The median number of days with limited activity was the same (10 days) for both genders.

A significant difference was noted in one aspect of the *Participation (D6)* domain—men significantly more often (63%) than women (35%) rated the time spent on health issues as severe (*p* = 0.047). Men also slightly more often reported a greater impact of health on their emotional state and a higher level of burden on their family due to their health problems, although these differences did not reach significance.

In summary, the analysis generally showed a comparable level of disability between women and men in most evaluated areas, with isolated differences in specific aspects of social participation and relationship building. Gender does not appear to have a significant impact on the overall level of disability as measured by the WHODAS 2.0 questionnaire in the studied patient sample.

### 3.7. Analysis of the Association between the Selected ICF Core Set Parameters and WHODAS 2.0

To verify the relationship between the selected codes in the *Activities and Participation* domain of the International Classification of Functioning, Disability and Health (ICF) and selected elements of the WHODAS 2.0 questionnaire, a correlation analysis was conducted. The aim was to identify the relationships between the level of dysfunction and the difficulties encountered by patients in various life domains defined by the ICF.

WHODAS 2.0 has a direct link with the ICF and is based on the biopsychosocial model, derived from specific ICF categories. This unique feature distinguishes WHODAS 2.0 from other instruments measuring functioning and disability. It is a standardized tool that directly relates to the *Activities and Participation* domain of the ICF questionnaire [14].

Table 13 presents the results of the Spearman correlation analysis, which allows for the assessment of the strength and direction of the relationship between ordinal variables. 

The analysis included elements of the WHODAS 2.0 questionnaire paired with ICF codes (*Activities and Participation* domain).

#### 3.7.1. Domain 2: Mobility 

Significant correlations were observed between mobility limitations and patients’ daily functioning. The ICF *Performance* qualifier for *Changing basic body position (d410)* showed a moderate correlation with WHODAS 2.0’s *Standing up from sitting down? (D2.2)* (*rho* = 0.38; *p* = 0.007), suggesting that difficulties with basic body movements during position changes may exacerbate overall mobility problems.

For the ICF code *Moving around in different locations (d460)*, both the *Performance* and *Capacity* qualifiers showed a stronger correlation with WHODAS 2.0’s *Moving around inside your home? (D2.3)* (*rho* = 0.32; *p* = 0.020 and *rho* = 0.47; *p* = 0.001), indicating that the ability to walk is a key element affecting independence in this domain. The same ICF code, the *Moving around in different locations (d460) Capacity* qualifier, also correlated with WHODAS 2.0’s *Getting out of your home? (D2.4)* (*rho* = 0.33; *p* = 0.019).

#### 3.7.2. Domain 3: Self-Care 

ICF codes related to self-care, such as the *Washing oneself (d510) Capacity* qualifier with WHODAS 2.0’s *Washing your whole body? (D3.1)* (*rho* = 0.33, *p* = 0.021) and the *Dressing (d540) Performance* qualifier with WHODAS 2.0’s *Getting dressed? (D3.2)* (*rho* = 0.35, *p* = 0.013), showed moderate correlations, highlighting that impairments in these areas may exacerbate difficulties in daily functioning.

#### 3.7.3. Domain 4: Getting along with People

The results for the ICF code *Intimate relationships (d770)* did not indicate statistically significant correlations.

#### 3.7.4. Domains 5(1) and 5(2): Household Activities and Work or School Activities

In the domain of work and education, strong negative correlations (*rho* = −0.76 to −0.85; *p* < 0.001) were observed between the ICF codes *Acquiring, keeping, and terminating a job (d845)* and *Remunerative employment (d850)* for the *Performance* and *Capacity* qualifiers, and WHODAS 2.0 elements, indicating a significant impact of disability on these aspects of life.

#### 3.7.5. Domain 6: Participation 

Codes such as *Emotional functions (b152)* and *Recreation and leisure (d920)* did not show significant correlations, suggesting that additional factors may influence an individual’s participation in social life and recreation.

## 4. Discussion

Based on the conducted study aimed at assessing the functional profile and degree of disability in patients following thoracic surgery for lung cancer, we can conclude that the results partially confirm the presented hypotheses. The custom-developed extended set of the ICF proved to be an effective tool for the comprehensive description of the patients’ functional profiles, thereby confirming research Hypothesis 1. Conversely, the results indicating a correlation between the degree of disability assessed using the WHO DAS 2.0 questionnaire and the *Activity and Participation* domain of the comprehensive ICF Core Set, although not unequivocal, suggest a certain relationship, indicating partial confirmation of research Hypothesis 2.

### 4.1. Analysis of the Comprehensive ICF Core Set Results for Lung Cancer Patients

Through three rounds of the Delphi consensus process, aiming to achieve agreement among interdisciplinary specialists evaluating the relevance of selected ICF classification codes to describe the functional profile of lung cancer patients post-surgery, a comprehensive ICF Core Set was developed, ultimately comprising 77 codes. The analysis identified twenty-three codes related to the *Body Function* domain, eight codes related to the *Body Structures* domain, twenty-five codes related to the *Activity and Participation* domain, and twenty-one codes related to the *Environmental Factors* domain. Our study, based on the biopsychosocial model of human functioning, aimed to develop an ICF Core Set for lung cancer to improve clinical practice and rehabilitation effectiveness. Currently, the WHO has developed only seven brief and comprehensive Core Sets for cardiovascular and respiratory conditions: Core Sets for Obstructive Pulmonary Diseases, Obesity, Diabetes Mellitus, Stroke, Chronic Ischaemic Heart Disease, Cardiopulmonary Conditions in post-acute care, and Cardiopulmonary Conditions in acute care [25]. There is still no proposed Core Set dedicated to lung cancer patients. We hope our study will serve as an impetus for developing classifications for this patient group.

Our findings highlight the crucial importance of psychological and emotional aspects in the context of surgical interventions, such as a lobectomy, reflected in selected ICF codes included in the *Body Function* domain, such as *Energy and drive functions (b130), Sleep functions (b134), Emotional functions (b152),* and *Range of emotion (b1522).* The necessity of incorporating a wide range of emotional and psychological functions in the care of post-lung resection patients stems from frequently observed issues in this area, including sleep disturbances and even depressive episodes despite effective treatment [26].

### 4.2. Analysis of Emotional and Psychological Function Codes

Analyzing the *Body Function* domain codes related to emotional and psychological functions in our study, we observe that both men and women most frequently indicated mild or moderate impairment. These results are consistent with the literature. According to research by Linares-Moya et al. [27], preoperative psychological distress is associated with greater symptomatic burden, poorer health status, and sleep quality one year after lung resection. This suggests that early psychological interventions could have significant implications for improving overall functioning and adaptation post-surgery [27].

### 4.3. Pain Sensation Codes in Post-Surgical Lung Cancer Patients

This study also focused on the most critical functions related to pain sensation in post-surgical lung cancer patients. Codes at the second, third, and fourth levels of the ICF classification related to pain, such as *Sensation of pain (b280), Pain in the body part (b2801)*, and *Pain in the chest (b28011)*, are essential for understanding the pain experiences of lobectomy patients. Both men and women showed moderate impairment in these codes. The assessment of pain in relation to the ICF classification, using the recommended ICF Rehabilitation Core Set (ICF-RCS), was analyzed in another study on lung cancer [28]. It demonstrated that the code *Sensation of pain (b280)* after surgery was similar for both genders, with moderate impairment in both women (86.96%) and men (85.19%). The correlations between the Laitinen pain scale and the code *Sensation of pain (b280)* in the ICF were strong and positive, confirming the consistency of these measurement tools. This is corroborated by our current study.

Research by Kajiyama et al. [29] also emphasizes the significance of pain management as a key element in improving the quality of life for post-lung cancer surgery patients. Intense postoperative pain can significantly impact the rehabilitation process and return to normal functioning [29]. Appropriate pain management strategies can contribute to shorter hospital stays and a reduced risk of chronic pain, aligning with the biopsychosocial model of human functioning.

### 4.4. Voice Function Codes Post-Lung Resection Surgery

In the medical literature, the importance of voice functions in the postoperative context for lung resection patients is increasingly highlighted. Reports indicate that mediastinal lymph node dissection allows for a precise pathological assessment of the disease stage but may lead to severe complications such as bleeding, chylothorax, atrial fibrillation, and recurrent laryngeal nerve (RLN) or phrenic nerve paralysis. A certain degree of hoarseness caused by RLN paralysis may be unavoidable during lymph node dissection in primary lung cancer surgery. Hoarseness is a well-known and representative complication of upper mediastinal lymph node dissection in NSCLC [30]. Therefore, our custom extended ICF set included the code *Voice functions (b310).* Interestingly, in our study, both genders rated *Voice functions (b310)* as having no impairment. 

In the context of heart-related functions, recognizing changes in the structure and function of the heart after lung resection surgery is crucial. Our study results for the code *Heart functions (b410)* showed that a higher percentage of men (55.56%) compared to women (26.09%) showed no impairment, while more women (43.48%) than men (18.52%) experienced mild impairment. These differences did not reach statistical significance (*p* = 0.115). A study by Chen et al. [31] utilized tissue Doppler echocardiography (TDI) to assess cardiac changes before and after surgery in a group of 43 patients. The results showed that although certain structural parameters of the heart, such as the ascending aorta width and the anteroposterior diameter of the left atrium, significantly changed, cardiac functions remained stable, confirming that TDI is an effective tool for monitoring post-lung resection patients. These findings are crucial for understanding how surgical interventions impact patients’ cardiac profiles and can help optimize postoperative management [31].

### 4.5. Hematological and Immunological Function Codes Post-Surgery

Analyzing the impact of surgical techniques on hematological and immunological functions is essential in the context of oncological interventions in early-stage NSCLC patients. A study conducted by Pu Q et al. in 2013 [32], comparing acute inflammatory responses and immunosuppression following a lobectomy via video-assisted thoracoscopy (VATS) and posterolateral thoracotomy (PLT), provided significant evidence of the less invasive nature and more favorable immunological profile of the VATS procedure. The analysis showed that VATS patients lost significantly less blood than PLT patients, which in itself may contribute to reduced complication risk and faster recovery. Additionally, serum amyloid A (SAA) levels, an acute-phase inflammatory marker, were significantly lower in VATS patients, suggesting a lesser inflammatory burden. This is crucial for oncology patients, where any additional burden can affect overall health and prognosis. Furthermore, less immunosuppression was observed in the VATS group, especially with higher CD8+ T cell levels compared to the PLT group. CD8+ T cells play a central role in the immune response to tumors, so their higher levels may translate to better tumor control and lower recurrence risk [32]. In our study, the distribution of impairment levels for the code *Structure of the immune system (s420)* was similar between both genders and was classified as mild impairment, and the differences did not reach statistical significance.

### 4.6. Respiratory Function Codes Pre- and Post-Lung Resection Surgery

In the context of respiratory functions in patients undergoing lung cancer surgery, evaluating and monitoring both pre- and post-surgery are significant. Regarding ICF codes, such as *Respiration functions (b440)*, *Respiration rate (b4400)*, *Respiratory muscle functions (b445)*, *Functions of the diaphragm (b4451)*, *Additional respiratory functions (b450)*, *Exercise tolerance functions (b455),* and *Sensations associated with cardiovascular and respiratory functions (b460)*, studies provide valuable insights into the long-term effects of surgery. For example, a study by Onodera et al. [33] analyzed predicted and actual respiratory function in patients post-lobectomy for lung cancer, highlighting differences between short- and long-term respiratory function outcomes. Patients experienced a noticeable decline in both lung vital capacity (VC) and forced expiratory volume for one second (FEV1.0) in both the short and long term post-surgery, although long-term outcomes were better than those observed immediately post-surgery. This suggests that respiratory functions, though initially decreased, can improve over time, which is essential for planning postoperative rehabilitation [33].

In our study, a trend was observed in the code *Additional respiratory functions (b450)*, where a higher percentage of men (11.11%) than women (0.00%) showed no impairment, while a higher percentage of women (56.52%) compared to men (37.04%) experienced mild impairment. The analysis of average patient responses in the *Body Function* domain showed that pain issues caused moderate impairment for patients with regard to the *Sensation of pain (b280)*, particularly *Pain in the chest (b28011)*, *Respiration functions (b440), Respiratory muscle functions (b445)*, *Exercise tolerance functions (b455)*, and *Muscle endurance functions (b740).*

Therefore, in the context of respiratory functions described in the ICF, integrating preoperative and postoperative assessments of respiratory functions and utilizing advanced predictive techniques, such as perfusion scintigraphy, is not only feasible but also recommended. This approach allows for a better understanding and management of the long-term effects of surgery on the patients’ respiratory systems, which can contribute to improved quality of life and the effectiveness of medical interventions [34].

### 4.7. Nutrition and Metabolism Function Codes Post-Surgery

In the context of nutritional and metabolic functions, understanding the impact of weight loss post-lung cancer surgery on patient prognosis is crucial. Data analysis from various studies highlights how weight loss affects health outcomes in patients undergoing a radical lobectomy. A study by Nakada et al. [35] showed that weight loss in the 6 to 12 months post-surgery period is associated with poor overall survival and disease-free survival, suggesting the need for continuous monitoring and nutritional interventions to improve patient prognosis [35].

Additionally, a study published by Moreno et al. [36] indicates that the nutritional status and inflammatory state of NSCLC patients did not significantly impact the risk of postoperative complications, suggesting that indicators, like BMI or inflammatory markers alone, may not be sufficient as independent predictors of postoperative outcomes [36].

According to the recommendations of the European Society for Clinical Nutrition and Metabolism, the DAIL study highlighted that a significant portion of advanced NSCLC patients suffer from malnutrition and require specialized dietary intervention, emphasizing the importance of early nutritional status diagnosis to prevent further health deterioration [37].

These findings underscore that maintaining body weight, as indicated by the code *Weight maintenance functions (b530)* in the comprehensive ICF Core Set post-lung cancer surgery, is a crucial factor influencing patient prognosis. Appropriate nutritional strategies and nutritional status monitoring are essential for improving treatment outcomes and quality of life for patients. Weight loss is a significant risk indicator and should be managed by a multidisciplinary team, including dietitians, oncologists, and surgeons, consistent with the latest oncology care standards. In our study, the distribution of impairment levels for the code *Weight maintenance functions (b530)* was similar between both genders and assessed as no, mild, or moderate impairment, with differences not reaching statistical significance.

### 4.8. Muscle Function and Mobility Codes Post-Surgery

In the context of muscle and movement-related functions, it is essential to comprehensively understand the impact of surgical treatment for lung cancer on muscle strength and endurance, as well as overall patient mobility. Research by Burtin et al. [38] provides significant insights into the relationship between lower limb muscle function and exercise tolerance in post-lung resection patients. These data indicate that isometric strength and endurance of the quadriceps muscle are significant predictors of both peak oxygen uptake (VO2peak) and peak work rate (Wpeak), emphasizing the role of muscle strength and endurance in optimizing rehabilitation outcomes post-surgery [38].

Additionally, a systematic review by Granger et al. [39] identifies barriers and facilitators of physical activity in lung cancer patients. The results highlight the need to consider multidimensional factors, such as symptoms, comorbidities, and mood, which may influence the ability and motivation of patients to engage in physical activity. Understanding these barriers is crucial for creating effective rehabilitation programs that support maintaining and improving muscle function and mobility [39].

These studies collectively illustrate the complex relationship between muscle functions and overall health and quality of life for post-lung cancer surgery patients. The importance of these functions in the context of ICF codes, such as joint mobility, muscle strength, muscle endurance, and sensations related to muscles and movement functions, is invaluable for effective rehabilitation and postoperative management.

### 4.9. Activity and Participation Domain According to the ICF

The role of physical activity and its impact on various aspects of life for post-lung cancer surgery patients is discussed in the context of the *Activity and Participation* domain according to the ICF. Various studies highlight the significance of early mobilization and long-term physical exercise in improving health outcomes and quality of life for patients. In a study by Cavalheri and Granger [40], the importance of early postoperative physical activity is emphasized, indicating that early mobilization in the hospital is a key element of the ERAS (Enhanced Recovery After Surgery) clinical pathway. It is noted that mobilizing patients within 24 h post-surgery brings benefits, such as reduced hospital stay and decreased morbidity [40].

Furthermore, a study by Solberg Nes et al. [41] in 2012 highlights the long-term impact of physical activity on the quality of life for lung cancer survivors. It was found that changes in physical activity levels from diagnosis to long-term follow-up were associated with changes in overall quality of life, symptom control, and reduced pain and fatigue. These results emphasize the need for implementing interventions aimed at increasing physical activity among this population [41].

These studies together illustrate that both early postoperative physical activity and maintaining long-term physical activity are crucial for improving functioning and quality of life for post-lung cancer surgery patients. Integrating these practices into care plans can significantly impact clinical outcomes and patient satisfaction. However, it requires the involvement of a multidisciplinary team, including rehabilitation specialists and physiotherapists, to provide patients with the necessary support and resources for active participation in social life and daily activities according to their individual needs.

An important aspect of participation for lung cancer patients, as shown in our study, was employment. The codes related to employment—*Acquiring, keeping, and terminating a job (d845)* and *Remunerative employment (d850)*—were rated the worst, with severe difficulty in the *Performance* qualifier. This indicates significant limitations in this area, which, according to the respondents, is influenced by their health condition.

### 4.10. Environmental Factors Domain According to the ICF

In the context of the *Environmental Factors* domain according to the ICF, understanding how various environmental factors influence the health and functioning of post-lung cancer surgery patients is crucial. Numerous studies show how important elements such as air quality, access to health services, and social support are in the treatment and recovery process. Our study in the *Environmental Factors* domain showed that only the code *Atmospheric pressure (e2252)* was rated as a mild barrier. Other codes were considered facilitators by the respondents. Complete facilitators included *Drugs (e1101), Immediate family (e310)*, and *Health professionals (e355)*. Patients with post-surgical procedures for lung cancer might rate *Atmospheric pressure (e2252)* as a minor barrier for several reasons: reduced lung capacity (lung surgery, especially resection, can lead to decreased lung volume and respiratory efficiency. Changes in atmospheric pressure, particularly drops associated with worsening weather, can cause respiratory discomfort, shortness of breath, and general malaise); postoperative pain (chest pain post-surgery can exacerbate with atmospheric pressure changes, leading to greater discomfort and limitation of activity); and changes in sensory perception (after surgery and during recovery, patients might be more sensitive to environmental changes, including atmospheric pressure variations. What was previously unnoticed can now be felt as discomfort) [42,43,44].

A study by Gu K. et al. [45] focused on analyzing the impact of the urban environment on lung cancer incidence, highlighting the correlation between factors such as land use, traffic, and availability of public spaces and increased lung cancer incidence in the Chinese city of Bengbu. Seasonal changes, such as the greater influence of spring and winter, significantly impacted cancer incidence, emphasizing the need to consider these factors when planning preventive and educational activities [45].

These studies shed light on the importance of the broader environment, including economic, social, and ecological factors, in combating lung cancer. They confirm that, in addition to advanced medical care, creating conditions conducive to health and well-being through improving air quality, the equitable distribution of health services, and raising awareness of the environmental impact on health is equally important. Such knowledge and actions are crucial for developing effective health and environmental strategies that support patients in their fight against the disease. The presented findings align with our results and highlight the importance of a holistic approach for the patient, encompassing both medical interventions and psychological support. Developing an ICF set for lung cancer, based on the biopsychosocial model, is a significant step toward more personalized and effective clinical practice and rehabilitation.

#### WHODAS 2.0 Data Analysis

The analysis of data from the WHODAS 2.0 questionnaire revealed some differences in the level of functioning between women and men in the studied group of lung cancer patients, with a few notable exceptions. In most evaluated domains, these differences did not reach statistical significance. 

In the *Cognition (D1)* domain, both women and men most frequently reported no problems or only mild difficulties in concentration, memory, problem solving, learning, and understanding speech. Similarly, in the *Mobility (D2)*, *Self-care (D3)*, and *Getting along (D4)* domains, the differences between genders were small and not statistically significant. The only significant difference concerned the question *Making new friends? (D4.4),* where a higher percentage of men (37%) than women (8.7%) reported no difficulty in forming new friendships (*p* = 0.031). In the *Life activities (D5)* domain, women more often reported no problems performing household tasks and responsibilities, although these differences were not statistically significant. A significant difference was noted in the *Participation (D6)* aspect, where men more often rated their time spent on health issues as severe (63% vs. 35%, *p* = 0.047). Men also slightly more frequently reported a greater impact of health on their emotional state and a higher level of burden on their family due to their health problems, although these differences did not reach significance. To understand the cause of these results, it is necessary to consider several factors.

*Socio-cultural roles and social support:* One key factor may be the difference in social roles and cultural expectations of women and men. Traditionally, men may have more support in forming social contacts and engaging in social activities, which may explain the higher percentage of men reporting no difficulty in making new friends. Women may be more burdened with household responsibilities and family care, affecting their ability to engage in new relationships.

*Perception of health and emotional well-being:* Differences in health perception and emotional burden may also play a role. Men more frequently reported spending significant time on health issues, which may stem from a greater tendency to report health problems and a greater emotional burden related to the disease. Women may be more inclined to minimize their health problems or prioritize other responsibilities over personal health.

*Biological differences and adaptation to disease:* Biological differences between genders can influence how patients experience and adapt to the disease. Hormonal and physiological differences can affect symptom perception and treatment responses, leading to differences in daily functioning. Men and women may react differently to pain, fatigue, and other symptoms associated with lung cancer and its treatment. 

The correlation analysis between selected codes from the *Activity and Participation* domain of the ICF and WHODAS 2.0 results showed significant relationships in various domains. In the *Mobility (D2)* domain, mobility limitations significantly impacted patients’ daily functioning. *Changing basic body position (d410)* correlated with difficulties in standing up from sitting (D2.2, *rho* = 0.38, *p* = 0.007). *Moving around in different locations (d460)* was a key element influencing patient independence, reflected in strong correlations with moving around inside the home (D2.3, *rho* = 0.32, *p* = 0.020 and *rho* = 0.47, *p* = 0.001) and getting out of the home (D2.4, rho = 0.33, *p* = 0.019). In the *Self-care (D3)* domain, ICF codes related to self-care, such as *Washing oneself (d510)* and *Dressing (d540)*, showed moderate correlations with corresponding questions from WHODAS 2.0, emphasizing that difficulties in these areas can exacerbate problems in daily functioning. In the work and education domain, strong negative correlations (*rho* = −0.76 to −0.85, *p* = 0.001) between ICF codes related to employment (d845, d850) and WHODAS 2.0 results indicate a significant impact of disability on these life aspects. Conversely, in the *Participation (D6)* domain, the lack of significant correlations for codes related to *Emotional functions (b152)* and *Recreation and leisure (d920)* suggests that other factors may influence participation in social life and recreation.

In summary, our results indicate that differences in the level of functioning between genders are mostly small and not statistically significant. However, there are specific areas, such as forming new friendships and the time spent on health issues, where men and women differ significantly. Additionally, correlations between *Activity and Participation* domain ICF codes and WHODAS 2.0 results highlight the crucial importance of mobility and self-care in the daily functioning of lung cancer patients. These differences may stem from a combination of socio-cultural, biological, and socio-economic contexts influencing how patients experience and cope with the disease. Further research should consider these factors to better understand their impact on patient functioning and develop more personalized approaches to healthcare.

### 4.11. Limitations

The conducted study, aimed at assessing the functional profile and degree of disability in patients following thoracic surgery for lung cancer, showed a partial confirmation of the presented hypotheses. However, its limitations stem from several significant factors: a relatively small sample size (*n* = 50) limits the statistical power and representativeness of the results, the single-center nature of this study may not reflect the diversity of clinical settings, and the lack of a control group prevents comparisons with other methods. Additionally, patient selection may have influenced the homogeneity of the group, and the subjective nature of the Delphi method and limitations of the WHODAS 2.0 tool may affect the objectivity of the results. The omission of a wide range of environmental and social factors that may influence the results also constitutes a significant limitation of this study. Furthermore, the occurrence of postoperative complications was not considered, which could be a significant variable affecting the results. The analysis also does not present the clinical data qualifying patients for surgery. Additionally, the observation period may be insufficient to capture long-term outcomes and the full impact of rehabilitation on functional status and disability. All these aspects should be considered when interpreting the results and planning future research in this field.

## 5. Conclusions

This study aimed to assess the functional profile and degree of disability in lung cancer patients post-thoracic surgery using the comprehensive ICF Core Set and WHODAS 2.0. Our results confirm the effectiveness of the ICF Core Set in evaluating various aspects of patient functioning, supporting the biopsychosocial model.

Data analysis from WHODAS 2.0 revealed some gender differences in functioning, with men showing slightly better results in forming new relationships and time spent on health issues. These differences, although not statistically significant, may be influenced by socio-cultural, biological, and socio-economic factors. Partial correlations between the degree of disability assessed using WHODAS 2.0 and the *Activity and Participation* domain of the ICF Core Set support the study’s hypotheses. Significant correlations, particularly in the *Mobility (D2)* and *Self-care (D3)* domains, highlight the importance of these areas in daily functioning and suggest the need for coordinated care and support in rehabilitation.

This study successfully developed an ICF Core Set for lung cancer, encompassing 77 codes related to body functions, structures, activity, participation, and environmental factors, demonstrating its utility in clinical practice and rehabilitation. Future research should involve larger, more diverse samples and control groups to validate these findings.

In summary, the ICF Core Set and WHODAS 2.0 are valuable tools for improving the assessment and rehabilitation of lung cancer patients, emphasizing the need for comprehensive and personalized care.

## Figures and Tables

**Table 1:** Qualifier coding convention in the Body Functions domain of the ICF classification.

Qualifier	Impairment Level	The Scale of the Problem	
xxx.0	NO impairment	(none, absent, negligible, …)	0–4%
xxx.1	MILD impairment	(slight, low, …)	5–24%
xxx.2	MODERATE impairment	(medium, fair, …)	25–49%
xxx.3	SEVERE impairment	(high, extreme, …)	50–95%
xxx.4	COMPLETE impairment	(total, …)	96–100%
xxx.8	not specified		
xxx.9	not applicable		

**Table 2:** Coding convention for the first qualifier in the Body Structures domain of the ICF classification.

Qualifier	Impairment Level	The Scale of the Problem	
xxx.0	NO impairment	(none, absent, negligible, …)	0–4%
xxx.1	MILD impairment	(slight, low, …)	5–24%
xxx.2	MODERATE impairment	(medium, fair, …)	25–49%
xxx.3	SEVERE impairment	(high, extreme, …)	50–95%
xxx.4	COMPLETE impairment	(total, …)	96–100%
xxx.8	not specified		
xxx.9	not applicable		

**Table 3:** Coding convention for the second and third qualifiers in the Body Structures domain of the ICF classification.

Second Qualifier	Nature
xxx.x0	no change in structure
xxx.x1	total absence
xxx.x2	partial absence
xxx.x3	additional part
xxx.x4	aberrant dimensions
xxx.x5	discontinuity
xxx.x6	deviating position
xxx.x7	qualitative changes in structure, including accumulation of fluid
	not specified
xxx.x8	not applicable
xxx.x9	
**Third Qualifier**	**Localization**
xxx.x0	more than one region
xxx.x1	right
xxx.x2	left
xxx.x3	both sides
xxx.x4	front
xxx.x5	back
xxx.x6	proximal
xxx.x7	distal
xxx.x8	not specified
xxx.x9	not applicable

**Table 4:** Coding convention for performance and capacity qualifiers in the Activities and Participation domain of the ICF classification.

Qualifier	Difficulty Level	The Scale of the Problem	
xxx.0	NO difficulty	(none, absent, negligible, …)	0–4%
xxx.1	MILD difficulty	(slight, low, …)	5–24%
xxx.2	MODERATE difficulty	(medium, fair, …)	25–49%
xxx.3	SEVERE difficulty	(high, extreme, …)	50–95%
xxx.4	COMPLETE difficulty	(total, …)	96–100%
xxx.8	not specified		
xxx.9	not applicable		

**Table 5:** Qualifier coding convention in the Environmental Factors domain of the ICF classification.

Qualifier	Barrier/Facilitator		The Scale of the Problem
xxx.0	NO barrier	(none, absent, negligible, …)	0–4%
xxx.1	MILD barrier	(slight, low, …)	5–24%
xxx.2	MODERATE barrier	(medium, fair, …)	25–49%
xxx.3	SEVERE barrier	(high, extreme, …)	50–95%
xxx.4	COMPLETE barrier	(total, …)	96–100%
xxx.+0	NO facilitator	(none, absent, negligible, …)	0–4%
xxx.+1	MILD facilitator	(slight, low, …)	5–24%
xxx.+2	MODERATE facilitator	(medium, fair, …)	25–49%
xxx.+3	SUBSTANTIAL facilitator	(high, extreme, …)	50–95%
xxx.+4	COMPLETE facilitator	(total, …)	96–100%
xxx.8	not specified		
xxx.9	not applicable		

**Table 6:** WHODAS 2.0 questionnaire item scoring scheme.

None	Mild	Moderate	Severe	Extreme or Cannot Do
1	2	3	4	5

**Table 7:** Distribution of metric and clinical data overall and by sex.

Characteristic	*N*	Overall ^a^	Sex		*p* ^c^
			Female, *n*_1_ = 23 ^a^	Male, *n*_2_ = 27 ^a^	
Age [years]	50	63.00 (57.25, 68.50)	60.00 (56.50, 65.00)	65.00 (61.00, 70.00)	0.070
Smoking	50	34.00 (68.00%) ^b^	13.00 (56.52%) ^b^	21.00 (77.78%) ^b^	0.108 ^d^
BMI [kg/m^2^]	50	24.60 (22.81, 28.01)	24.80 (21.94, 27.58)	24.28 (22.89, 27.59)	1.000 ^d^
Location of the lesions	50				**0.047** ^d^
Left lung		25.00 (50.00%) ^b^	8.00 (34.78%) ^b^	17.00 (62.96%) ^b^	
Right lung		25.00 (50.00%) ^b^	15.00 (65.22%) ^b^	10.00 (37.04%) ^b^	
Lung re-expansion time [days]	50	5.00 (4.00, 5.00)	4.00 (4.00, 5.00)	5.00 (4.00, 6.00)	0.265
Past cancer episode	50	17.00 (34.00%) ^b^	10.00 (43.48%) ^b^	7.00 (25.93%) ^b^	0.192 ^d^

^a^ Mdn (Q1, Q3); ^b^ *n* (%); ^c^ Wilcoxon rank-sum test; ^d^ Pearson’s chi-squared test.

**Table 8:** Delphi consensus assessment results for the Body Functions domain.

ICF Code		ICF Body Functions Category Title	Inter-Rater Agreement M ± SD		
2nd	3rd/4rd		Round 1	Round 2	Round 3
**b110**		Consciousness functions	0.40 ± 0.50	0.24 ± 0.44	0.00 ± 0.00
**b114**		Orientation functions	0.24 ± 0.44	0.12 ± 0.33	0.00 ± 0.00
**b130**		Energy and drive functions	1.00 ± 0.00	1.00 ± 0.00	1.00 ± 0.00
**b134**		Sleep functions	1.00 ± 0.00	1.00 ± 0.00	1.00 ± 0.00
**b152**		Emotional functions	1.00 ± 0.00	1.00 ± 0.00	1.00 ± 0.00
	**b1522**	Range of emotion	1.00 ± 0.00	1.00 ± 0.00	1.00 ± 0.00
**b280**		Sensation of pain	1.00 ± 0.00	1.00 ± 0.00	1.00 ± 0.00
	**b2801**	Pain in body part	1.00 ± 0.00	1.00 ± 0.00	1.00 ± 0.00
	**b28011**	Pain in chest	1.00 ± 0.00	1.00 ± 0.00	1.00 ± 0.00
**b310**		Voice functions	1.00 ± 0.00	1.00 ± 0.00	1.00 ± 0.00
**b410**		Heart functions	1.00 ± 0.00	1.00 ± 0.00	1.00 ± 0.00
**b415**		Blood vessel functions	0.56 ± 0.51	0.36 ± 0.49	0.00 ± 0.00
**b420**		Blood pressure functions	0.48 ± 0.51	0.16 ± 0.37	0.00 ± 0.00
**b430**		Hematological system functions	1.00 ± 0.00	1.00 ± 0.00	1.00 ± 0.00
**b435**		Immunological system functions	1.00 ± 0.00	1.00 ± 0.00	1.00 ± 0.00
**b440**		Respiration functions	1.00 ± 0.00	1.00 ± 0.00	1.00 ± 0.00
	**b4400**	Respiration rate	1.00 ± 0.00	1.00 ± 0.00	1.00 ± 0.00
**b445**		Respiratory muscle functions	1.00 ± 0.00	1.00 ± 0.00	1.00 ± 0.00
	**b4451**	Functions of the diaphragm	1.00 ± 0.00	1.00 ± 0.00	1.00 ± 0.00
**b450**		Additional respiratory functions	1.00 ± 0.00	1.00 ± 0.00	1.00 ± 0.00
**b455**		Exercise tolerance functions	1.00 ± 0.00	1.00 ± 0.00	1.00 ± 0.00
**b460**		Sensations associated with cardiovascular and respiratory functions	1.00 ± 0.00	1.00 ± 0.00	1.00 ± 0.00
**b510**		Ingestion functions	0.36 ± 0.49	0.00 ± 0.00	0.00 ± 0.00
**b530**		Weight maintenance functions	1.00 ± 0.00	1.00 ± 0.00	1.00 ± 0.00
**b545**		Water, mineral, and electrolyte balance functions	0.56 ± 0.51	0.28 ± 0.46	0.00 ± 0.00
**b610**		Urinary excretory functions	0.56 ± 0.51	0.20 ± 0.41	0.00 ± 0.00
**b710**		Mobility of joint functions	1.00 ± 0.00	1.00 ± 0.00	1.00 ± 0.00
**b730**		Muscle power functions	1.00 ± 0.00	1.00 ± 0.00	1.00 ± 0.00
**b740**		Muscle endurance functions	1.00 ± 0.00	1.00 ± 0.00	1.00 ± 0.00
**b780**		Sensations related to muscles and movement functions	1.00 ± 0.00	1.00 ± 0.00	1.00 ± 0.00
**b820**		Repair functions of the skin	0.12 ± 0.33	0.00 ± 0.00	0.00 ± 0.00

**Table 9:** Delphi consensus assessment results for the Body Structures domain.

ICF Code		ICF Body Structures Category Title	Inter-Rater Agreement M ± SD		
2nd	3rd/4rd		Round 1	Round 2	Round 3
**s410**		Structure of the cardiovascular system	1.00 ± 0.00	1.00 ± 0.00	1.00 ± 0.00
**s420**		Structure of the immune system	1.00 ± 0.00	1.00 ± 0.00	1.00 ± 0.00
**s430**		Structure of the respiratory system	1.00 ± 0.00	1.00 ± 0.00	1.00 ± 0.00
	**s4301**	Lungs	1.00 ± 0.00	1.00 ± 0.00	1.00 ± 0.00
	**s4303**	Muscles of respiration	1.00 ± 0.00	1.00 ± 0.00	1.00 ± 0.00
**s710**		Structure of the head and neck region	0.12 ± 0.33	0.00 ± 0.00	0.00 ± 0.00
**s720**		Structure of the shoulder region	1.00 ± 0.00	1.00 ± 0.00	1.00 ± 0.00
**s760**		Structure of the trunk	1.00 ± 0.00	1.00 ± 0.00	1.00 ± 0.00
	**s76001**	Thoracic vertebral column	1.00 ± 0.00	1.00 ± 0.00	1.00 ± 0.00
**s810**		Structure of areas of skin	0.24 ± 0.44	0.00 ± 0.00	0.00 ± 0.00

**Table 10:** Delphi consensus assessment results for the Activities and Participation domain.

ICF Code		ICF Activities and Participation Category Title	Inter-Rater Agreement M ± SD		
2nd	3rd/4rd		Round 1	Round 2	Round 3
**d230**		Carrying out daily routine	1.00 ± 0.00	1.00 ± 0.00	1.00 ± 0.00
**d240**		Handling stress and other psychological demands	1.00 ± 0.00	1.00 ± 0.00	1.00 ± 0.00
**d330**		Speaking	1.00 ± 0.00	1.00 ± 0.00	1.00 ± 0.00
**d410**		Changing basic body position	1.00 ± 0.00	1.00 ± 0.00	1.00 ± 0.00
**d415**		Maintaining a body position	0.52 ± 0.51	0.24 ± 0.44	0.00 ± 0.00
**d420**		Transferring oneself	0.36 ± 0.49	0.00 ± 0.00	0.00 ± 0.00
**d430**		Lifting and carrying objects	1.00 ± 0.00	1.00 ± 0.00	1.00 ± 0.00
**d445**		Hand and arm use	1.00 ± 0.00	1.00 ± 0.00	1.00 ± 0.00
**d450**		Walking	1.00 ± 0.00	1.00 ± 0.00	1.00 ± 0.00
**d455**		Moving around	1.00 ± 0.00	1.00 ± 0.00	1.00 ± 0.00
**d460**		Moving around in different locations	1.00 ± 0.00	1.00 ± 0.00	1.00 ± 0.00
**d465**		Moving around using equipment	0.52 ± 0.51	0.16 ± 0.37	0.00 ± 0.00
**d470**		Using transportation	1.00 ± 0.00	1.00 ± 0.00	1.00 ± 0.00
**d475**		Driving	1.00 ± 0.00	1.00 ± 0.00	1.00 ± 0.00
	**d4750**	Driving human-powered transportation	1.00 ± 0.00	1.00 ± 0.00	1.00 ± 0.00
**d510**		Washing oneself	1.00 ± 0.00	1.00 ± 0.00	1.00 ± 0.00
**d520**		Caring for body parts	0.68 ± 0.48	0.32 ± 0.48	0.00 ± 0.00
**d540**		Dressing	1.00 ± 0.00	1.00 ± 0.00	1.00 ± 0.00
**d570**		Looking after one’s health	1.00 ± 0.00	1.00 ± 0.00	1.00 ± 0.00
	**d5701**	Managing diet and fitness	1.00 ± 0.00	1.00 ± 0.00	1.00 ± 0.00
**d620**		Acquisition of goods and services	1.00 ± 0.00	1.00 ± 0.00	1.00 ± 0.00
**d640**		Doing housework	1.00 ± 0.00	1.00 ± 0.00	1.00 ± 0.00
**d650**		Caring for household objects	1.00 ± 0.00	1.00 ± 0.00	1.00 ± 0.00
**d660**		Assisting others	0.64 ± 0.49	0.24 ± 0.44	0.00 ± 0.00
**d720**		Complex interpersonal interactions	1.00 ± 0.00	1.00 ± 0.00	1.00 ± 0.00
**d760**		Family relationships	1.00 ± 0.00	1.00 ± 0.00	1.00 ± 0.00
**d770**		Intimate relationships	1.00 ± 0.00	1.00 ± 0.00	1.00 ± 0.00
**d845**		Acquiring, keeping, and terminating a job	1.00 ± 0.00	1.00 ± 0.00	1.00 ± 0.00
**d850**		Remunerative employment	1.00 ± 0.00	1.00 ± 0.00	1.00 ± 0.00
**d910**		Community life	0.48 ± 0.51	0.12 ± 0.33	0.00 ± 0.00
**d920**		Recreation and leisure	1.00 ± 0.00	1.00 ± 0.00	1.00 ± 0.00

**Table 11:** Delphi consensus assessment results for the Environmental Factors domain.

ICF Code		ICF Environmental Factors Category Title	Inter-Rater Agreement M ± SD		
2nd	3rd/4rd		Round 1	Round 2	Round 3
**e110**		Products or substances for personal consumption	1.00 ± 0.00	1.00 ± 0.00	1.00 ± 0.00
	**e1101**	Drugs	1.00 ± 0.00	1.00 ± 0.00	1.00 ± 0.00
**e115**		Products and technology for personal use in daily living	1.00 ± 0.00	1.00 ± 0.00	1.00 ± 0.00
**e120**		Products and technology for personal indoor and outdoor mobility and transportation	1.00 ± 0.00	1.00 ± 0.00	1.00 ± 0.00
**e150**		Design, construction, and building products and technology of buildings for public use	0.28 ± 0.46	0.00 ± 0.00	0.00 ± 0.00
**e155**		Design, construction, and building products and technology of buildings for private use	1.00 ± 0.00	1.00 ± 0.00	1.00 ± 0.00
**e225**		Climate	1.00 ± 0.00	1.00 ± 0.00	1.00 ± 0.00
	**e2252**	Atmospheric pressure	1.00 ± 0.00	1.00 ± 0.00	1.00 ± 0.00
**e245**		Time-related changes	0.44 ± 0.51	0.20 ± 0.41	0.00 ± 0.00
	**e2450**	Day/night cycles	0.00 ± 0.00	0.00 ± 0.00	0.00 ± 0.00
**e250**		Sound	0.16 ± 0.37	0.00 ± 0.00	0.00 ± 0.00
**e260**		Air quality	1.00 ± 0.00	1.00 ± 0.00	1.00 ± 0.00
**e310**		Immediate family	1.00 ± 0.00	1.00 ± 0.00	1.00 ± 0.00
**e320**		Friends	1.00 ± 0.00	1.00 ± 0.00	1.00 ± 0.00
**e340**		Personal care providers and personal assistants	1.00 ± 0.00	1.00 ± 0.00	1.00 ± 0.00
**e350**		Domesticated animals	1.00 ± 0.00	1.00 ± 0.00	1.00 ± 0.00
**e355**		Health professionals	1.00 ± 0.00	1.00 ± 0.00	1.00 ± 0.00
**e410**		Individual attitudes of immediate family members	1.00 ± 0.00	1.00 ± 0.00	1.00 ± 0.00
**e420**		Individual attitudes of friends	1.00 ± 0.00	1.00 ± 0.00	1.00 ± 0.00
**e450**		Individual attitudes of health professionals	1.00 ± 0.00	1.00 ± 0.00	1.00 ± 0.00
**e460**		Societal attitudes	0.36 ± 0.49	0.16 ± 0.37	0.00 ± 0.00
**e540**		Transportation services, systems, and policies	1.00 ± 0.00	1.00 ± 0.00	1.00 ± 0.00
**e555**		Associations and organizational services, systems, and policies	0.36 ± 0.49	0.00 ± 0.00	0.00 ± 0.00
**e570**		Social security services, systems, and policies	0.44 ± 0.51	0.00 ± 0.00	0.00 ± 0.00
**e575**		General social support services, systems, and policies	1.00 ± 0.00	1.00 ± 0.00	1.00 ± 0.00
**e580**		Health services, systems, and policies	1.00 ± 0.00	1.00 ± 0.00	1.00 ± 0.00
	**e5800**	Health services	1.00 ± 0.00	1.00 ± 0.00	1.00 ± 0.00
**e585**		Education and training services, systems, and policies	0.20 ± 0.41	0.00 ± 0.00	0.00 ± 0.00
**e590**		Labor and employment services, systems, and policies	1.00 ± 0.00	1.00 ± 0.00	1.00 ± 0.00

**Table 12:** Characteristics of the WHODAS 2.0 disability assessment scores by sex.

Characteristic	*N*	Sex		*p* ^c^
		Female *n*_1_ = 23 ^a^	Male *n*_2_ = 27 ^a^	
Domain 1: Cognition				
**D1.1: Concentrating on doing something for ten minutes?**	50			0.878
none		6.00 (26.09%)	7.00 (25.93%)	
mild		15.00 (65.22%)	15.00 (55.56%)	
moderate		2.00 (8.70%)	4.00 (14.81%)	
severe		0.00 (0.00%)	1.00 (3.70%)	
**D1.2: Remembering to do important things?**	50			1.000
none		6.00 (26.09%)	6.00 (22.22%)	
mild		15.00 (65.22%)	18.00 (66.67%)	
moderate		2.00 (8.70%)	3.00 (11.11%)	
**D1.3: Analyzing and finding solutions to problems in day-to-day life?**	50			0.475
none		17.00 (73.91%)	16.00 (59.26%)	
mild		6.00 (26.09%)	9.00 (33.33%)	
moderate		0.00 (0.00%)	2.00 (7.41%)	
**D1.4: Learning a new task, for example, learning how to get to a new place?**	50			0.695
none		5.00 (21.74%)	5.00 (18.52%)	
mild		16.00 (69.57%)	16.00 (59.26%)	
moderate		2.00 (8.70%)	5.00 (18.52%)	
severe		0.00 (0.00%)	1.00 (3.70%)	
**D1.5: Generally understanding what people say?**	50			0.614
none		22.00 (95.65%)	24.00 (88.89%)	
mild		1.00 (4.35%)	3.00 (11.11%)	
**D1.6: Starting and maintaining a** **conversation?**	50			
none		23.00 (100.00%)	27.00 (100.00%)	
Domain 2: Mobility				
**D2.1: Standing for long periods, such as 30 min?**	50			0.449
none		12.00 (52.17%)	9.00 (33.33%)	
mild		6.00 (26.09%)	11.00 (40.74%)	
moderate		5.00 (21.74%)	6.00 (22.22%)	
severe		0.00 (0.00%)	1.00 (3.70%)	
**D2.2: Standing up from sitting down?**	50			1.000
none		19.00 (82.61%)	22.00 (81.48%)	
mild		4.00 (17.39%)	4.00 (14.81%)	
moderate		0.00 (0.00%)	1.00 (3.70%)	
**D2.3: Moving around inside your home?**	50			0.493
none		23.00 (100.00%)	25.00 (92.59%)	
mild		0.00 (0.00%)	2.00 (7.41%)	
**D2.4: Getting out of your home?**	50			0.240
none		23.00 (100.00%)	23.00 (85.19%)	
mild		0.00 (0.00%)	3.00 (11.11%)	
moderate		0.00 (0.00%)	1.00 (3.70%)	
**D2.5: Walking a long distance, such as a kilometer [or equivalent]?**	50			0.958
none		5.00 (21.74%)	4.00 (14.81%)	
mild		13.00 (56.52%)	16.00 (59.26%)	
moderate		5.00 (21.74%)	6.00 (22.22%)	
severe		0.00 (0.00%)	1.00 (3.70%)	
Domain 3: Self-care				
**D3.1: Washing your whole body?**	50			0.783
none		13.00 (56.52%)	12.00 (44.44%)	
mild		7.00 (30.43%)	11.00 (40.74%)	
moderate		3.00 (13.04%)	3.00 (11.11%)	
severe		0.00 (0.00%)	1.00 (3.70%)	
**D3.2: Getting dressed?**	50			0.948
none		15.00 (65.22%)	15.00 (55.56%)	
mild		7.00 (30.43%)	9.00 (33.33%)	
moderate		1.00 (4.35%)	2.00 (7.41%)	
severe		0.00 (0.00%)	1.00 (3.70%)	
**D3.3: Eating?**	50			0.632
none		14.00 (60.87%)	13.00 (48.15%)	
mild		8.00 (34.78%)	11.00 (40.74%)	
moderate		1.00 (4.35%)	3.00 (11.11%)	
**D3.4: Staying by yourself for a few days?**	50			0.494
none		14.00 (60.87%)	13.00 (48.15%)	
mild		9.00 (39.13%)	12.00 (44.44%)	
moderate		0.00 (0.00%)	2.00 (7.41%)	
Domain 4: Getting along with people				
**D4.1: Dealing with people you do not know?**	50			1.000
none		3.00 (13.04%)	4.00 (14.81%)	
mild		19.00 (82.61%)	22.00 (81.48%)	
moderate		1.00 (4.35%)	1.00 (3.70%)	
**D4.2: Maintaining a friendship?**	50			0.643 ^d^
none		13.00 (56.52%)	17.00 (62.96%)	
mild		10.00 (43.48%)	10.00 (37.04%)	
**D4.3: Getting along with people who are close to you?**	50			1.000
none		21.00 (91.30%)	25.00 (92.59%)	
mild		2.00 (8.70%)	2.00 (7.41%)	
**D4.4: Making new friends?**	50			0.031
none		2.00 (8.70%)	10.00 (37.04%)	
mild		20.00 (86.96%)	17.00 (62.96%)	
moderate		1.00 (4.35%)	0.00 (0.00%)	
**D4.5: Sexual activities?**	50			0.885
none		12.00 (52.17%)	12.00 (44.44%)	
mild		10.00 (43.48%)	14.00 (51.85%)	
moderate		1.00 (4.35%)	1.00 (3.70%)	
Domain 5: Life activities				
**D5.1: Taking care of your household responsibilities?**	50			0.345
none		15.00 (65.22%)	13.00 (48.15%)	
mild		8.00 (34.78%)	12.00 (44.44%)	
moderate		0.00 (0.00%)	2.00 (7.41%)	
**D5.2: Doing your most important household tasks well?**	50			0.278
none		16.00 (69.57%)	13.00 (48.15%)	
mild		6.00 (26.09%)	11.00 (40.74%)	
moderate		1.00 (4.35%)	3.00 (11.11%)	
**D5.3: Getting all the household work done that you needed to do?**	50			0.658
none		9.00 (39.13%)	7.00 (25.93%)	
mild		12.00 (52.17%)	16.00 (59.26%)	
moderate		2.00 (8.70%)	4.00 (14.81%)	
**D5.4: Getting your household work done as quickly as needed?**	50			0.173
none		5.00 (21.74%)	1.00 (3.70%)	
mild		15.00 (65.22%)	22.00 (81.48%)	
moderate		3.00 (13.04%)	4.00 (14.81%)	
**D5.01: In the past 30 days, how many days did you reduce or** **completely miss household work because of your health** **condition?**	50	10.00 (5.00, 20.00) ^b^	10.00 (10.00, 25.00) ^b^	0.119 ^e^
**D5.5: Your day-to-day work/school?**	50			0.898
none		10.00 (43.48%)	12.00 (44.44%)	
mild		9.00 (39.13%)	8.00 (29.63%)	
moderate		4.00 (17.39%)	6.00 (22.22%)	
severe		0.00 (0.00%)	1.00 (3.70%)	
**D5.6: Doing your most important work/school tasks well?**	50			0.923
none		10.00 (43.48%)	12.00 (44.44%)	
mild		9.00 (39.13%)	11.00 (40.74%)	
moderate		4.00 (17.39%)	3.00 (11.11%)	
severe		0.00 (0.00%)	1.00 (3.70%)	
**D5.7: Getting all the work done that you need to do?**	50			0.712
none		10.00 (43.48%)	12.00 (44.44%)	
mild		5.00 (21.74%)	3.00 (11.11%)	
moderate		8.00 (34.78%)	11.00 (40.74%)	
severe		0.00 (0.00%)	1.00 (3.70%)	
**D5.8: Getting your work done as quickly as needed?**	50			0.482
none		10.00 (43.48%)	12.00 (44.44%)	
mild		0.00 (0.00%)	1.00 (3.70%)	
moderate		12.00 (52.17%)	10.00 (37.04%)	
severe		1.00 (4.35%)	4.00 (14.81%)	
**D5.9: Have you had to work at a lower level because of a health condition?**	50			0.945 ^d^
not applicable (0)		10.00 (43.48%)	12.00 (44.44%)	
Yes (2) No (1)		13.00 (56.52%) 0.00 (0.00%)	15.00 (55.56%) 0.00 (0.00%)	
**D5.10: Did you earn less money as a result of a health condition?**	50			1.000
not applicable (0)		10.00 (43.48%)	12.00 (44.44%)	
No (1)		1.00 (4.35%)	2.00 (7.41%)	
Yes (2)		12.00 (52.17%)	13.00 (48.15%)	
**D5.02: In the past 30 days, how many days did you miss work for** **half a day or more because of your health condition?**	50	10.00 (0.00, 10.00) ^b^	10.00 (0.00, 10.00) ^b^	0.967 ^e^
Domain 6: Participation				
**D6.1: How much of a problem did you have joining community activities (for example, festivities, religious or other activities) in the same way as anyone else can?**	50			0.387
none		13.00 (56.52%)	16.00 (59.26%)	
mild		8.00 (34.78%)	11.00 (40.74%)	
moderate		2.00 (8.70%)	0.00 (0.00%)	
**D6.2: How much of a problem did you have because of barriers or hindrances in the world around you?**	50			0.641
none		5.00 (21.74%)	3.00 (11.11%)	
mild		14.00 (60.87%)	19.00 (70.37%)	
moderate		4.00 (17.39%)	5.00 (18.52%)	
**D6.3: How much of a problem did you have living with dignity because of the attitudes and actions of others?**	50			0.115
none		10.00 (43.48%)	10.00 (37.04%)	
mild		10.00 (43.48%)	17.00 (62.96%)	
moderate		3.00 (13.04%)	0.00 (0.00%)	
**D6.4: How much time did you spend on your health condition or its consequences?**	50			0.047 ^d^
moderate		15.00 (65.22%)	10.00 (37.04%)	
severe		8.00 (34.78%)	17.00 (62.96%)	
**D6.5: How much have you been emotionally affected by your health condition?**	50			0.231
moderate		13.00 (56.52%)	10.00 (37.04%)	
severe		10.00 (43.48%)	15.00 (55.56%)	
extreme		0.00 (0.00%)	2.00 (7.41%)	
**D6.6: How much has your health been a drain on the financial resources of you or your family?**	50			0.387
mild		1.00 (4.35%)	0.00 (0.00%)	
moderate		15.00 (65.22%)	15.00 (55.56%)	
severe		7.00 (30.43%)	12.00 (44.44%)	
**D6.7: How much of a problem did your family have because of your health problems?**	50			0.065
moderate		16.00 (69.57%)	11.00 (40.74%)	
severe		7.00 (30.43%)	15.00 (55.56%)	
extreme		0.00 (0.00%)	1.00 (3.70%)	
**D6.8: How much of a problem did you have in doing things by yourself for relaxation or pleasure?**	50			0.153
none		12.00 (52.17%)	9.00 (33.33%)	
mild		10.00 (43.48%)	18.00 (66.67%)	
moderate		1.00 (4.35%)	0.00 (0.00%)	

^a^ *n* (%); ^b^ Mdn (Q1, Q3); ^c^ Fisher’s exact test; ^d^ Pearson’s chi-squared test; ^e^ Wilcoxon rank-sum test.

**Table 13:** Analysis of the association between the WHO DAS 2.0 and ICF.

WHO DAS 2.0	ICF Code	Performance		Capacity	
		*rho*	*p*	*rho*	*p*
Domain 2: Mobility					
**D2.2**	**d410**	0.38	**0.007**	0.24	0.097
**D2.3**	**d460**	0.32	**0.020**	0.47	**0.001**
**D2.4**	**d460**	0.15	0.295	0.33	**0.019**
**D2.5**	**d450**	0.19	0.193	0.18	0.203
Domain 3: Self-care					
**D3.1**	**d510**	0.15	0.294	0.33	**0.021**
**D3.2**	**d540**	0.35	**0.013**	0.28	0.050
**D3.4**	**d510**	0.10	0.478	0.23	0.110
**D3.4**	**d540**	0.24	0.098	0.23	0.102
**D3.4**	**d570**	−0.18	0.215	−0.11	0.446
**D3.4**	**d5701**	−0.16	0.277	−0.21	0.145
**D3.4**	**d620**	0.03	0.861	0.08	0.547
**D3.4**	**d640**	0.03	0.854	−0.08	0.577
**D3.4**	**d650**	0.10	0.489	−0.13	0.370
Domain 4: Getting along with people					
**D4.1**	**d720 ^1^**	n/a	n/a	n/a	n/a
**D4.2**	**d720**	n/a	n/a	n/a	n/a
**D4.3**	**d760 ^2^**	n/a	n/a	n/a	n/a
**D4.3**	**d770**	−0.17	0.229	−0.14	0.340
**D4.4**	**d720**	n/a	n/a	n/a	n/a
**D4.5**	**d770**	0.03	0.852	0.12	0.400
Domain 5(1): Household activities					
**D5.1**	**d640**	0.27	0.062	0.01	0.927
**D5.1**	**d650**	0.18	0.220	0.13	0.396
**D5.2**	**d640**	0.25	0.075	0.02	0.917
**D5.2**	**d650**	0.14	0.343	0.13	0.376
**D5.3**	**d640**	0.23	0.100	0.02	0.900
**D5.3**	**d650**	0.08	0.575	0.07	0.614
**D5.4**	**d640**	0.26	0.066	−0.03	0.819
**D5.4**	**d650**	0.15	0.311	0.08	0.571
Domain 5(2): Work or school activities					
**D5.5**	**d845**	−0.82	**<0.001**	−0.78	**<0.001**
**D5.5**	**d850**	−0.77	**<0.001**	−0.77	**<0.001**
**D5.6**	**d845**	−0.77	**<0.001**	−0.76	**<0.001**
**D5.6**	**d850**	−0.76	**<0.001**	−0.78	**<0.001**
**D5.7**	**d845**	−0.82	**<0.001**	−0.81	**<0.001**
**D5.7**	**d850**	−0.81	**<0.001**	−0.83	**<0.001**
**D5.8**	**d845**	−0.85	**<0.001**	−0.84	**<0.001**
**D5.8**	**d850**	−0.82	**<0.001**	−0.84	**<0.001**
Domain 6: Participation					
**D6.5**	**b152**	0.16	0.244	-	-
**D6.8**	**d920**	0.08	0.605	0.12	0.425

^1^ The feature exhibits zero variance (all values are zero); ^2^ the feature exhibits zero variance; n/a—not applicable.

## Data Availability

The measurement data used to support the findings of this study are available from the corresponding author upon request.

## Supplementary Materials

The following supporting information can be downloaded at https://www.mdpi.com/article/10.3390/cancers16122281/s1, Supplementary Materials S1 Table S1: Characteristics of Body Functions domain codes assessment results in the study sample by sex; Supplementary Materials S2 Table S2: Characteristics of *Body Structures* domain codes assessment results in the study sample by sex; Supplementary Materials S3 Table S3: Characteristics of Activities and Participation domain codes—Performance qualifier assessment results in the study sample by sex; Supplementary Materials S4 Table S4: Characteristics of Activities and Participation domain codes—Capacity qualifier assessment results in the study sample by sex; Supplementary Materials S5 Table S5: Characteristics of Environmental Factors domain codes assessment results in the study sample by sex.
