# Supplementary material for: Functional and Disability Outcomes in NSCLC Patients Post-Lobectomy Undergoing Pulmonary Rehabilitation: A Biopsychosocial Approach
Source: Cancers (Basel). 2024 Jun 20;16(12):2281. doi: 10.3390/cancers16122281 (PMC11201694; doi:10.3390/cancers16122281)
Supplement: Supplementary file 1 [file cancers-16-02281-s001.zip › Supplementary Material S1.pdf]

Supplementary Material

Analysis of the distribution of the results of the ICF questionnaire – *Body Function domain*

Table S1. Characteristics of *Body Functions* domain codes assessment results in the study sample by sex.

Characteristic	*N*	*Sex*		p^b
		Female, $n_1 = 23^a$	*Male,* $n_2 = 27^a$	
**Energy and drive functions (b130)**	50			
no impairment		1,00 (4,35%)	9,00 (33,33%)	**0,011**
mild impairment		17,00(73,91%)	16,00 (59,26%)	0,276[d]
moderate impairment		5,00 (21,74%)	2,00 (7,41%)	0,146
**Sleep functions (b134)**	50			0,668
no impairment		1,00 (4,35%)	3,00 (11,11%)	
mild impairment		20,00 (86,96%)	20,00 (74,07%)	
moderate impairment		2,00 (8,70%)	4,00 (14,81%)	
**Emotional functions (b152)**	50			0,475
no impairment		1,00 (4,35%)	3,00 (11,11%)	
mild impairment		12,00 (52,17%)	12,00 (44,44%)	
moderate impairment		7,00 (30,43%)	11,00 (40,74%)	
severe impairment		3,00 (13,04%)	1,00 (3,70%)	
**Range of emotion (b1522)**	50			0,330
no impairment		1,00 (4,35%)	5,00 (18,52%)	
mild impairment		19,00 (82,61%)	20,00 (74,07%)	
moderate impairment		3,00 (13,04%)	2,00 (7,41%)	
**Sensation of pain (b280)**	50			0,905
mild impairment		0,00 (0,00%)	1,00 (3,70%)	
moderate impairment		19,00 (82,61%)	23,00 (85,19%)	
severe impairment		3,00 (13,04%)	2,00 (7,41%)	
complete impairment		1,00 (4,35%)	1,00 (3,70%)	
**Pain in body part (b2801)**	50			0,672
no impairment		2,00 (8,70%)	3,00 (11,11%)	
mild impairment		14,00 (60,87%)	19,00 (70,37%)	
moderate impairment		6,00 (26,09%)	4,00 (14,81%)	
severe impairment		1,00 (4,35%)	0,00 (0,00%)	
complete impairment		0,00 (0,00%)	1,00 (3,70%)	
**Pain in chest (b28011)**	50			0,909
mild impairment		1,00 (4,35%)	0,00 (0,00%)	
moderate impairment		18,00 (78,26%)	23,00 (85,19%)	
severe impairment		3,00 (13,04%)	3,00 (11,11%)	
complete impairment		1,00 (4,35%)	1,00 (3,70%)	
**Voice functions (b310)**	50			0,075
no impairment		18,00 (78,26%)	19,00 (70,37%)	
mild impairment		5,00 (21,74%)	3,00 (11,11%)	
moderate impairment		0,00 (0,00%)	5,00 (18,52%)	
**Heart functions (b410)**	50			0,115
no impairment		6,00 (26,09%)	15,00 (55,56%)	
mild impairment		10,00 (43,48%)	5,00 (18,52%)	
moderate impairment		5,00 (21,74%)	3,00 (11,11%)	

severe impairment		2,00 (8,70%)	3,00 (11,11%)	
complete impairment		0,00 (0,00%)	1,00 (3,70%)	
**Haematological system functions (b430)**	50			0,968[c]
no impairment		11,00 (47,83%)	12,00 (44,44%)	
mild impairment		7,00 (30,43%)	9,00 (33,33%)	
moderate impairment		5,00 (21,74%)	6,00 (22,22%)	
**Immunological system functions (b435)**	50			0,709
no impairment		5,00 (21,74%)	9,00 (33,33%)	
mild impairment		15,00 (65,22%)	15,00 (55,56%)	
moderate impairment		3,00 (13,04%)	3,00 (11,11%)	
**Respiration functions (b440)**	50			0,938
no impairment		0,00 (0,00%)	1,00 (3,70%)	
mild impairment		3,00 (13,04%)	3,00 (11,11%)	
moderate impairment		17,00 (73,91%)	21,00 (77,78%)	
severe impairment		3,00 (13,04%)	2,00 (7,41%)	
**Respiration rate (b4400)**	50			1,000
no impairment		20,00 (86,96%)	23,00 (85,19%)	
mild impairment		2,00 (8,70%)	2,00 (7,41%)	
moderate impairment		1,00 (4,35%)	2,00 (7,41%)	
**Respiratory muscle functions (b445)**	50			0,926
no impairment		0,00 (0,00%)	1,00 (3,70%)	
mild impairment		4,00 (17,39%)	6,00 (22,22%)	
moderate impairment		18,00 (78,26%)	19,00 (70,37%)	
severe impairment		1,00 (4,35%)	1,00 (3,70%)	
**Functions of the diaphragm (b4451)**	50			0,570
no impairment		17,00 (73,91%)	20,00 (74,07%)	
mild impairment		6,00 (26,09%)	5,00 (18,52%)	
moderate impairment		0,00 (0,00%)	2,00 (7,41%)	
**Additional respiratory functions (b450)**	50			0,189
no impairment		0,00 (0,00%)	3,00 (11,11%)	
mild impairment		13,00 (56,52%)	10,00 (37,04%)	
moderate impairment		9,00 (39,13%)	13,00 (48,15%)	
severe impairment		1,00 (4,35%)	0,00 (0,00%)	
complete impairment		0,00 (0,00%)	1,00 (3,70%)	
**Exercise tolerance functions (b455)**	50			0,372
moderate impairment		19,00 (82,61%)	21,00 (77,78%)	
severe impairment		3,00 (13,04%)	6,00 (22,22%)	
complete impairment		1,00 (4,35%)	0,00 (0,00%)	
**Sensations associated with cardiovascular and respiratory functions (b460)**	50			0,936
no impairment		0,00 (0,00%)	1,00 (3,70%)	
mild impairment		14,00 (60,87%)	17,00 (62,96%)	
moderate impairment		8,00 (34,78%)	8,00 (29,63%)	
severe impairment		1,00 (4,35%)	1,00 (3,70%)	
**Weight maintenance functions (b530)**	50			0,856
no impairment		11,00 (47,83%)	14,00 (51,85%)	
mild impairment		5,00 (21,74%)	7,00 (25,93%)	
moderate impairment		5,00 (21,74%)	6,00 (22,22%)	
severe impairment		1,00 (4,35%)	0,00 (0,00%)	
complete impairment		1,00 (4,35%)	0,00 (0,00%)	
**Mobility of joint functions (b710)**	50			0,932

no impairment		7,00 (30,43%)	11,00 (40,74%)	
mild impairment		12,00 (52,17%)	12,00 (44,44%)	
moderate impairment		2,00 (8,70%)	2,00 (7,41%)	
severe impairment		2,00 (8,70%)	2,00 (7,41%)	
**Muscle power functions (b730)**	50			0,838
no impairment		3,00 (13,04%)	2,00 (7,41%)	
mild impairment		9,00 (39,13%)	13,00 (48,15%)	
moderate impairment		11,00 (47,83%)	11,00 (40,74%)	
severe impairment		0,00 (0,00%)	1,00 (3,70%)	
**Muscle endurance functions (b740)**	50			0,374
mild impairment		5,00 (21,74%)	2,00 (7,41%)	
moderate impairment		17,00 (73,91%)	24,00 (88,89%)	
severe impairment		1,00 (4,35%)	1,00 (3,70%)	
**Sensations related to muscles and movement functions (b780)**	50			0,889
no impairment		4,00 (17,39%)	6,00 (22,22%)	
mild impairment		18,00 (78,26%)	19,00 (70,37%)	
moderate impairment		1,00 (4,35%)	2,00 (7,41%)	

[a] n (%)
[b] Fisher’s exact test
[c] Pearson's Chi-squared test
[d] Proportion test
