# Supplementary material for: Functional and Disability Outcomes in NSCLC Patients Post-Lobectomy Undergoing Pulmonary Rehabilitation: A Biopsychosocial Approach
Source: Cancers (Basel). 2024 Jun 20;16(12):2281. doi: 10.3390/cancers16122281 (PMC11201694; doi:10.3390/cancers16122281)
Supplement: Supplementary file 1 [file cancers-16-02281-s001.zip › Supplementary Material S3 and S4.pdf]

Supplementary Material

Analysis of the distribution of the results of the ICF questionnaire – *Activities and Participation domain*

Table S3. Characteristics of *Activities and Participation* domain codes – *Performance* qualifier sassessment results in the study sample by sex.

Characteristic	N	*Sex* *Female,* $n_1 = 23^a$	*Male,* $n_2 = 27^a$	p^b
**Carrying out daily routine (d230)**	50			0,109
no difficulty		2,00 (8,70%)	8,00 (29,63%)	
mild difficulty		20,00 (86,96%)	16,00 (59,26%)	
moderate difficulty		1,00 (4,35%)	3,00 (11,11%)	
**Handling stress and other psychological demands (d240)**	50			0,475
no difficulty		1,00 (4,35%)	3,00 (11,11%)	
mild difficulty		12,00 (52,17%)	12,00 (44,44%)	
complete difficulty		3,00 (13,04%)	1,00 (3,70%)	
moderate difficulty		7,00 (30,43%)	11,00 (40,74%)	
**Speaking (d330)**	50			0,561
no difficulty		19,00 (82,61%)	21,00 (77,78%)	
mild difficulty		4,00 (17,39%)	3,00 (11,11%)	
complete difficulty		0,00 (0,00%)	1,00 (3,70%)	
moderate difficulty		0,00 (0,00%)	2,00 (7,41%)	
**Changing basic body position (d410)**	50			0,711
no difficulty		20,00 (86,96%)	22,00 (81,48%)	
mild difficulty		3,00 (13,04%)	5,00 (18,52%)	
**Lifting and carrying objects (d430)**	50			1,000
no difficulty		6,00 (26,09%)	8,00 (29,63%)	
mild difficulty		14,00 (60,87%)	16,00 (59,26%)	
moderate difficulty		3,00 (13,04%)	3,00 (11,11%)	
**Hand and arm use (d445)**	50			0,687
no difficulty		15,00 (65,22%)	14,00 (51,85%)	
mild difficulty		7,00 (30,43%)	12,00 (44,44%)	
moderate difficulty		1,00 (4,35%)	1,00 (3,70%)	
**Walking (d450)**	50			1,000
no difficulty		23,00 (100,00%)	25,00 (92,59%)	
mild difficulty		0,00 (0,00%)	1,00 (3,70%)	
moderate difficulty		0,00 (0,00%)	1,00 (3,70%)	
**Moving around (d455)**	50			
not applicable		23,00 (100,00%)	27,00 (100,00%)	
**Moving around in different locations (d460)**	50			0,123[c]
no difficulty		19,00 (82,61%)	17,00 (62,96%)	
mild difficulty		4,00 (17,39%)	10,00 (37,04%)	
**Using transportation (d470)**	50			0,871
no difficulty		5,00 (21,74%)	8,00 (29,63%)	
mild difficulty		17,00 (73,91%)	18,00 (66,67%)	
moderate difficulty		1,00 (4,35%)	1,00 (3,70%)	

**Driving (d475)**	50			0,928
no difficulty		0,00 (0,00%)	1,00 (3,70%)	
mild difficulty		12,00 (52,17%)	13,00 (48,15%)	
moderate difficulty		7,00 (30,43%)	7,00 (25,93%)	
not applicable		4,00 (17,39%)	6,00 (22,22%)	
**Driving human-powered transportation (d4750)**	50			0,768
no difficulty		1,00 (4,35%)	1,00 (3,70%)	
mild difficulty		16,00 (69,57%)	15,00 (55,56%)	
complete difficulty		0,00 (0,00%)	1,00 (3,70%)	
moderate difficulty		6,00 (26,09%)	10,00 (37,04%)	
**Washing oneself (d510)**	50			0,606
no difficulty		0,00 (0,00%)	2,00 (7,41%)	
mild difficulty		20,00 (86,96%)	21,00 (77,78%)	
moderate difficulty		3,00 (13,04%)	4,00 (14,81%)	
**Dressing (d540)**	50			1,000
no difficulty		1,00 (4,35%)	1,00 (3,70%)	
mild difficulty		18,00 (78,26%)	22,00 (81,48%)	
moderate difficulty		4,00 (17,39%)	4,00 (14,81%)	
**Looking after one's health (d570)**	50			0,538
mild difficulty		3,00 (13,04%)	7,00 (25,93%)	
complete difficulty		9,00 (39,13%)	8,00 (29,63%)	
moderate difficulty		11.00 (47.83%)	12,00 (44,44%)	
**Managing diet and fitness (d5701)**	50			0,895
no difficulty		0,00 (0,00%)	1,00 (3,70%)	
mild difficulty		6,00 (26,09%)	6,00 (22,22%)	
moderate difficulty		14,00 (60,87%)	18,00 (66,67%)	
severe difficulty		2,00 (8,70%)	2,00 (7,41%)	
complete difficulty		1,00 (4,35%)	0,00 (0,00%)	
**Acquisition of goods and services (d620)**	50			0,834
no difficulty		19,00 (82,61%)	23,00 (85,19%)	
mild difficulty		3,00 (13,04%)	4,00 (14,81%)	
moderate difficulty		1,00 (4,35%)	0,00 (0,00%)	
**Doing housework (d640)**	50			0,327
no difficulty		5,00 (21,74%)	3,00 (11,11%)	
mild difficulty		13,00 (56,52%)	20,00 (74,07%)	
moderate difficulty		5,00 (21,74%)	3,00 (11,11%)	
severe difficulty		0,00 (0,00%)	1,00 (3,70%)	
**Caring for household objects (d650)**	50			1,000
no difficulty		6,00 (26,09%)	6,00 (22,22%)	
mild difficulty		15,00 (65,22%)	18,00 (66,67%)	
moderate difficulty		2,00 (8,70%)	3,00 (11,11%)	
**Complex interpersonal interactions (d720)**	50			
no difficulty		23,00 (100,00%)	27,00 (100,00%)	
**Family relationships (d760)**	50			
no difficulty		23,00 (100,00%)	27,00 (100,00%)	
**Intimate relationships (d770**)	50			0,128
no difficulty		20,00 (86,96%)	17,00 (62,96%)	
mild difficulty		2,00 (8,70%)	8,00 (29,63%)	
moderate difficulty		1,00 (4,35%)	2,00 (7,41%)	

**Acquiring, keeping and terminating a job (d845)**	50			0,959
no difficulty		2,00 (8,70%)	4,00 (14,81%)	
mild difficulty		7,00 (30,43%)	6,00 (22,22%)	
moderate difficulty		3,00 (13,04%)	3,00 (11,11%)	
severe difficulty		0,00 (0,00%)	1,00 (3,70%)	
complete difficulty		1,00 (4,35%)	1,00 (3,70%)	
not applicable		10,00 (43,48%)	12,00 (44,44%)	
**Remunerative employment (d850)**	50			1,000
no difficulty		3,00 (13,04%)	3,00 (11,11%)	
mild difficulty		7,00 (30,43%)	7,00 (25,93%)	
moderate difficulty		2,00 (8,70%)	3,00 (11,11%)	
severe difficulty		0,00 (0,00%)	1,00 (3,70%)	
complete difficulty		1,00 (4,35%)	1,00 (3,70%)	
not applicable		10,00 (43,48%)	12,00 (44,44%)	
**Recreation and leisure (d920)**	50			0,287
no difficulty		11,00 (47,83%)	15,00 (55,56%)	
mild difficulty		11,00 (47,83%)	7,00 (25,93%)	
moderate difficulty		1,00 (4,35%)	3,00 (11,11%)	
severe difficulty		0,00 (0,00%)	2,00 (7,41%)	

[a] n (%)
[b] Fisher's exact test
[c] Pearson's Chi-squared test

Table S4. Characteristics of *Activities and Participation* domain codes – *Capacity* qualifier sassessment results in the study sample by sex.

Characteristic	N	*Sex* *Female,* $n_1 = 23^a$	*Male,* $n_2 = 27^a$	p^b
**Carrying out daily routine (d230)**	50			0,109
no difficulty		2,00 (8,70%)	8,00 (29,63%)	
mild difficulty		20,00 (86,96%)	16,00 (59,26%)	
moderate difficulty		1,00 (4,35%)	3,00 (11,11%)	
**Handling stress and other psychological demands (d240)**	50			0,618
no difficulty		1,00 (4,35%)	3,00 (11,11%)	
mild difficulty		14,00 (60,87%)	13,00 (48,15%)	
complete difficulty		2,00 (8,70%)	1,00 (3,70%)	
moderate difficulty		6,00 (26,09%)	10,00 (37,04%)	
**Speaking (d330)**	50			0,357
no difficulty		22,00 (95,65%)	22,00 (81,48%)	
mild difficulty		1,00 (4,35%)	4,00 (14,81%)	
moderate difficulty		0,00 (0,00%)	1,00 (3,70%)	
**Changing basic body position (d410)**	50			1,000
no difficulty		21,00 (91,30%)	25,00 (92,59%)	

mild difficulty		2,00 (8,70%)	2,00 (7,41%)	
**Lifting and carrying objects (d430)**	50			1,000
no difficulty		20,00 (86,96%)	24,00 (88,89%)	
mild difficulty		3,00 (13,04%)	3,00 (11,11%)	
**Hand and arm use (d445)**	50			1,000
no difficulty		22,00 (95,65%)	26,00 (96,30%)	
mild difficulty		1,00 (4,35%)	1,00 (3,70%)	
**Walking (d450)**	50			0,713
no difficulty		22,00 (95,65%)	26,00 (96,30%)	
mild difficulty		0,00 (0,00%)	1,00 (3,70%)	
moderate difficulty		1,00 (4,35%)	0,00 (0,00%)	
**Moving around (d455)**	50			
not applicable		23,00 (100,00%)	27,00 (100,00%)	
**Moving around in different locations (d460)**	50			0,713
no difficulty		22,00 (95,65%)	26,00 (96,30%)	
mild difficulty		0,00 (0,00%)	1,00 (3,70%)	
moderate difficulty		1,00 (4,35%)	0,00 (0,00%)	
**Using transportation (d470)**	50			0,276[c]
no difficulty		17,00 (73,91%)	16,00 (59,26%)	
mild difficulty		6,00 (26,09%)	11,00 (40,74%)	
**Driving (d475)**	50			0,640
no difficulty		9,00 (39,13%)	12,00 (44,44%)	
mild difficulty		8,00 (34,78%)	5,00 (18,52%)	
moderate difficulty		2,00 (8,70%)	4,00 (14,81%)	
not applicable		4,00 (17,39%)	6,00 (22,22%)	
**Driving human-powered transportation (d4750)**	50			0,568
no difficulty		15,00 (65,22%)	14,00 (51,85%)	
mild difficulty		6,00 (26,09%)	11,00 (40,74%)	
moderate difficulty		2,00 (8,70%)	2,00 (7,41%)	
**Washing oneself (d510)**	50			0,827[c]
no difficulty		16,00 (69,57%)	18,00 (66,67%)	
mild difficulty		7,00 (30,43%)	9,00 (33,33%)	
**Dressing (d540)**	50			0,781[c]
no difficulty		17,00 (73,91%)	19,00 (70,37%)	
mild difficulty		6,00 (26,09%)	8,00 (29,63%)	
**Looking after one's health (d570)**	50			0,507
no difficulty		1,00 (4,35%)	3,00 (11,11%)	
mild difficulty		6,00 (26,09%)	11,00 (40,74%)	
complete difficulty		4,00 (17,39%)	3,00 (11,11%)	
moderate difficulty		12,00 (52,17%)	10,00 (37,04%)	
**Managing diet and fitness (d5701)**	50			0,645
no difficulty		7,00 (30,43%)	7,00 (25,93%)	
mild difficulty		8,00 (34,78%)	14,00 (51,85%)	
moderate difficulty		6,00 (26,09%)	5,00 (18,52%)	
severe difficulty		2,00 (8,70%)	1,00 (3,70%)	
**Acquisition of goods and services (d620)**	50			0,725
no difficulty		21,00 (91,30%)	26,00 (96,30%)	
mild difficulty		1,00 (4,35%)	1,00 (3,70%)	
moderate difficulty		1,00 (4,35%)	0,00 (0,00%)	

**Doing housework (d640)**	50			0,543
no difficulty		14,00 (60,87%)	17,00 (62,96%)	
mild difficulty		9,00 (39,13%)	8,00 (29,63%)	
moderate difficulty		0,00 (0,00%)	2,00 (7,41%)	
**Caring for household objects (d650)**	50			1,000
no difficulty		19,00 (82,61%)	22,00 (81,48%)	
mild difficulty		4,00 (17,39%)	5,00 (18,52%)	
**Complex interpersonal interactions (d720)**	50			
no difficulty		23,00 (100,00%)	27,00 (100,00%)	
**Family relationships (d760)**	50			
no difficulty		23,00 (100,00%)	27,00 (100,00%)	
**Intimate relationships (d770**)	50			0,315
no difficulty		21,00 (91,30%)	20,00 (74,07%)	
mild difficulty		2,00 (8,70%)	5,00 (18,52%)	
moderate difficulty		0,00 (0,00%)	2,00 (7,41%)	
**Acquiring, keeping and terminating a job (d845)**	50			1,000
no difficulty		9,00 (39,13%)	9,00 (33,33%)	
mild difficulty		3,00 (13,04%)	4,00 (14,81%)	
moderate difficulty		0,00 (0,00%)	1,00 (3,70%)	
complete difficulty		1,00 (4,35%)	1,00 (3,70%)	
not applicable		10,00 (43,48%)	12,00 (44,44%)	
**Remunerative employment (d850)**	50			0,676
no difficulty		11,00 (47,83%)	9,00 (33,33%)	
mild difficulty		1,00 (4,35%)	4,00 (14,81%)	
moderate difficulty		0,00 (0,00%)	1,00 (3,70%)	
complete difficulty		1,00 (4,35%)	1,00 (3,70%)	
not applicable		10,00 (43,48%)	12,00 (44,44%)	
**Recreation and leisure (d920)**	50			0,161
no difficulty		11,00 (47,83%)	15,00 (55,56%)	
mild difficulty		12,00 (52,17%)	8,00 (29,63%)	
moderate difficulty		0,00 (0,00%)	3,00 (11,11%)	
severe difficulty		0,00 (0,00%)	1,00 (3,70%)	

[a] n (%)
[b] Fisher's exact test
[c] Pearson's Chi-squared test
