# Supplementary material for: Functional and Disability Outcomes in NSCLC Patients Post-Lobectomy Undergoing Pulmonary Rehabilitation: A Biopsychosocial Approach
Source: Cancers (Basel). 2024 Jun 20;16(12):2281. doi: 10.3390/cancers16122281 (PMC11201694; doi:10.3390/cancers16122281)
Supplement: Supplementary file 1 [file cancers-16-02281-s001.zip › Supplementary Material S5.pdf]

Supplementary Material

Analysis of the distribution of the results of the ICF questionnaire – *Environmental Factors domain*

Table S5. Characteristics of *Environmental Factors* domain codes assessment results in the study sample by sex.

Characteristic	N	*Sex*		p^b
		Female, $n_1 = 23^a$	*Male*, $n_2 = 27^a$	
**Products or substances for personal consumption (e110)**	50			0,375
complete facilitator		8,00 (34,78%)	12,00 (44,44%)	
substantial facilitator		11,00 (47,83%)	6,00 (22,22%)	
moderate facilitator		3,00 (13,04%)	7,00 (25,93%)	
mild facilitator		1,00 (4,35%)	2,00 (7,41%)	
**Drugs (e1101)**	50			1,000
complete facilitator		18,00 (78,26%)	22,00 (81,48%)	
substantial facilitator		5,00 (21,74%)	5,00 (18,52%)	
**Products and technology for personal use in daily living (e115)**	50			0,382
complete facilitator		8,00 (34,78%)	12,00 (44,44%)	
substantial facilitator		12,00 (52,17%)	8,00 (29,63%)	
moderate facilitator		2,00 (8,70%)	6,00 (22,22%)	
mild facilitator		1,00 (4,35%)	1,00 (3,70%)	
**Products and technology for personal indoor and outdoor mobility and transportation (e120)**	50			0,118
complete facilitator		8,00 (34,78%)	12,00 (44,44%)	
substantial facilitator		11,00 (47,83%)	5,00 (18,52%)	
moderate facilitator		3,00 (13,04%)	5,00 (18,52%)	
mild facilitator		1,00 (4,35%)	5,00 (18,52%)	
**Design, construction and building products and technology of buildings for private use (e155)**	50			0,123
no facilitator/barrier		1,00 (4,35%)	1,00 (3,70%)	
complete facilitator		7,00 (30,43%)	11,00 (40,74%)	
substantial facilitator		10,00 (43,48%)	4,00 (14,81%)	
moderate facilitator		4,00 (17,39%)	5,00 (18,52%)	
mild facilitator		1,00 (4,35%)	6,00 (22,22%)	
**Climate (e225)**	50			0,430
no facilitator/barrier		11,00 (47,83%)	13,00 (48,15%)	
mild barrier		2,00 (8,70%)	3,00 (11,11%)	
severe barrier		3,00 (13,04%)	1,00 (3,70%)	
modrate barrier		3,00 (13,04%)	8,00 (29,63%)	
mild facilitator		4,00 (17,39%)	2,00 (7,41%)	
**Atmospheric pressure (e2252)**	50			0,914
no facilitator/barrier		9,00 (39,13%)	9,00 (33,33%)	
mild barrier		5,00 (21,74%)	7,00 (25,93%)	
severe barrier		3,00 (13,04%)	2,00 (7,41%)	
modrate barrier		5,00 (21,74%)	8,00 (29,63%)	
mild facilitator		1,00 (4,35%)	1,00 (3,70%)	
**Air quality (e260)**	50			0,287

no facilitator/barrier		12,00 (52,17%)	14,00 (51,85%)	
mild barrier		3,00 (13,04%)	4,00 (14,81%)	
severe barrier		3,00 (13,04%)	0,00 (0,00%)	
modrate barrier		4,00 (17,39%)	8,00 (29,63%)	
complete facilitator		0,00 (0,00%)	1,00 (3,70%)	
mild facilitator		1,00 (4,35%)	0,00 (0,00%)	
**Immediate family (e310)**	50			0,667
complete facilitator		12,00 (52,17%)	16,00 (59,26%)	
substantial facilitator		11,00 (47,83%)	10,00 (37,04%)	
moderate facilitator		0,00 (0,00%)	1,00 (3,70%)	
**Friends (e320)**	50			0,962
no facilitator/barrier		2,00 (8,70%)	2,00 (7,41%)	
complete facilitator		9,00 (39,13%)	12,00 (44,44%)	
substantial facilitator		11,00 (47,83%)	11,00 (40,74%)	
moderate facilitator		1,00 (4,35%)	2,00 (7,41%)	
**Personal care providers and personal assistants (e340)**	50			0,362
no facilitator/barrier		11,00 (47,83%)	14,00 (51,85%)	
complete facilitator		2,00 (8,70%)	1,00 (3,70%)	
substantial facilitator		4,00 (17,39%)	6,00 (22,22%)	
moderate facilitator		6,00 (26,09%)	3,00 (11,11%)	
mild facilitator		0,00 (0,00%)	3,00 (11,11%)	
**Domesticated animals (e350)**	50			0,587
no facilitator/barrier		7,00 (30,43%)	5,00 (18,52%)	
complete facilitator		2,00 (8,70%)	1,00 (3,70%)	
substantial facilitator		6,00 (26,09%)	6,00 (22,22%)	
moderate facilitator		8,00 (34,78%)	13,00 (48,15%)	
mild facilitator		0,00 (0,00%)	2,00 (7,41%)	
**Health professionals (e355)**	50			0,880
complete facilitator		12,00 (52,17%)	15,00 (55,56%)	
substantial facilitator		11,00 (47,83%)	11,00 (40,74%)	
moderate facilitator		0,00 (0,00%)	1,00 (3,70%)	
**Individual attitudes of immediate family members (e410)**	50			0,958
complete facilitator		4,00 (17,39%)	3,00 (11,11%)	
substantial facilitator		12,00 (52,17%)	15,00 (55,56%)	
moderate facilitator		7,00 (30,43%)	8,00 (29,63%)	
mild facilitator		0,00 (0,00%)	1,00 (3,70%)	
**Individual attitudes of friends (e420)**	50			0,892
no facilitator/barrier		1,00 (4,35%)	1,00 (3,70%)	
complete facilitator		2,00 (8,70%)	1,00 (3,70%)	
substantial facilitator		12,00 (52,17%)	12,00 (44,44%)	
moderate facilitator		7,00 (30,43%)	11,00 (40,74%)	
mild facilitator		1,00 (4,35%)	2,00 (7,41%)	
**Individual attitudes of health professionals (e450)**	50			0,643
complete facilitator		3,00 (13,04%)	2,00 (7,41%)	
substantial facilitator		12,00 (52,17%)	14,00 (51,85%)	
moderate facilitator		7,00 (30,43%)	11,00 (40,74%)	
mild facilitator		1,00 (4,35%)	0,00 (0,00%)	

**Transportation services, systems and policies (e540)**	50			0,212
mild barrier		1,00 (4,35%)	0,00 (0,00%)	
no facilitator/barrier		0,00 (0,00%)	4,00 (14,81%)	
complete facilitator		1,00 (4,35%)	0,00 (0,00%)	
substantial facilitator		6,00 (26,09%)	4,00 (14,81%)	
moderate facilitator		5,00 (21,74%)	8,00 (29,63%)	
mild facilitator		10,00 (43,48%)	11,00 (40,74%)	
**General social support services, systems and policies (e575)**	50			0,457
mild barrier		0,00 (0,00%)	3,00 (11,11%)	
moderate barrier		1,00 (4,35%)	0,00 (0,00%)	
no facilitator/barrier		0,00 (0,00%)	1,00 (3,70%)	
complete facilitator		1,00 (4,35%)	0,00 (0,00%)	
substantial facilitator		3,00 (13,04%)	2,00 (7,41%)	
moderate facilitator		6,00 (26,09%)	7,00 (25,93%)	
mild facilitator		12,00 (52,17%)	14,00 (51,85%)	
**Health services, systems and policies (e580)**	50			0,895
mild barrier		2,00 (8,70%)	2,00 (7,41%)	
moderate barrier		1,00 (4,35%)	2,00 (7,41%)	
complete facilitator		1,00 (4,35%)	0,00 (0,00%)	
substantial facilitator		2,00 (8,70%)	2,00 (7,41%)	
moderate facilitator		7,00 (30,43%)	6,00 (22,22%)	
mild facilitator		10,00 (43,48%)	15,00 (55,56%)	
**Health services (e5800)**	50			0,968
mild barrier		3,00 (13,04%)	3,00 (11,11%)	
moderate barrier		0,00 (0,00%)	1,00 (3,70%)	
complete facilitator		1,00 (4,35%)	1,00 (3,70%)	
substantial facilitator		2,00 (8,70%)	2,00 (7,41%)	
moderate facilitator		8,00 (34,78%)	7,00 (25,93%)	
mild facilitator		9,00 (39,13%)	13,00 (48,15%)	
**Labour and employment services, systems and policies (e590)**	50			0,414
mild barrier		0,00 (0,00%)	1,00 (3,70%)	
no facilitator/barrier		2,00 (8,70%)	5,00 (18,52%)	
substantial facilitator		2,00 (8,70%)	2,00 (7,41%)	
moderate facilitator		8,00 (34,78%)	4,00 (14,81%)	
mild facilitator		11,00 (47,83%)	15,00 (55,56%)	

[a] n (%)

[b] Fisher's exact test
