# Supplementary material for: Functional and Disability Outcomes in NSCLC Patients Post-Lobectomy Undergoing Pulmonary Rehabilitation: A Biopsychosocial Approach
Source: Cancers (Basel). 2024 Jun 20;16(12):2281. doi: 10.3390/cancers16122281 (PMC11201694; doi:10.3390/cancers16122281)
Supplement: Supplementary file 1 [file cancers-16-02281-s001.zip › Supplementray Material S2.pdf]

Supplementary Material

Analysis of the distribution of the results of the ICF questionnaire – *Body Structures domain*

Table S2. Characteristics of *Body Structures* domain codes assessment results in the study sample by sex.

Characteristic	N	*Sex*		p^b
		Female, $n_1 = 23$[a]	*Male,* $n_2 = 27$[a]	
**Structure of cardiovascular system (s410)**	50			
no impairment		6,00 (26,09%)	15,00(55,56%)	**0,035**[c]
mild impairment		10,00(43,48%)	5,00 (18,52%)	0,055[c]
moderate impairment		6,00 (26,09%)	2,00 (7,41%)	0,072
severe impairment		1,00 (4,35%)	4,00 (14,81%)	0,218
complete impairment		0,00 (0,00%)	1,00 (3,70%)	0,351
**Structure of immune system (s420)**	50			0,243
no impairment		6,00 (26,09%)	11,00 (40,74%)	
mild impairment		15,00(65,22%)	15,00(55,56%)	
moderate impairment		2,00 (8,70%)	0,00 (0,00%)	
severe impairment		0,00 (0,00%)	1,00 (3,70%)	
**Structure of respiratory system (s430)**	50			0,814
mild impairment		1,00 (4,35%)	0,00 (0,00%)	
moderate impairment		20,00(86,96%)	24,00(88,89%)	
severe impairment		2,00 (8,70%)	3,00 (11,11%)	
**Lungs (s4301**)	50			0,194
moderate impairment		15,00(65,22%)	13,00(48,15%)	
severe impairment		7,00 (30,43%)	14,00(51,85%)	
complete impairment		1,00 (4,35%)	0,00 (0,00%)	
**Muscles of respiration (s4303)**	50			1,000
mild impairment		6,00 (26,09%)	8,00 (29,63%)	
moderate impairment		15,00(65,22%)	17,00(62,96%)	
severe impairment		2,00 (8,70%)	2,00 (7,41%)	
**Structure of shoulder region (s720)**	50			0,690
no impairment		13,00(56,52%)	11,00 (40,74%)	
mild impairment		8,00 (34,78%)	11,00 (40,74%)	
moderate impairment		2,00 (8,70%)	4,00 (14,81%)	
complete impairment		0,00 (0,00%)	1,00 (3,70%)	
**Structure of trunk (s760)**	50			1,000
no impairment		21,00(91,30%)	24,00(88,89%)	
mild impairment		2,00 (8,70%)	2,00 (7,41%)	
moderate impairment		0,00 (0,00%)	1,00 (3,70%)	
**Thoracic vertebral column (s76001)**	50			**0,038**
no impairment		19,00(82,61%)	26,00 (96,30%)	0,351[c]
mild impairment		4,00 (17,39%)	0,00 (0,00%)	**0,239**
moderate impairment		0,00 (0,00%)	1,00 (3,70%)	0,351

[a] n (%)
[b] Fisher's exact test
[c] Proportion test
